# SIRT2 alleviated renal fibrosis by deacetylating SMAD2 and SMAD3 in renal tubular epithelial cells

**DOI:** 10.1038/s41419-023-06169-1

**Published:** 2023-09-30

**Authors:** Shu Yang, Guangyan Yang, Xinyu Wang, Jiaqing Xiang, Lin Kang, Zhen Liang

**Affiliations:** 1 Department of Geriatrics, Shenzhen People’s Hospital (The Second Clinical Medical College of Jinan University & The First Affiliated Hospital of Southern University of Science and Technology), Shenzhen, China; 2 Guangdong Provincial Clinical Research Center for Geriatrics, Shenzhen Clinical Research Center for Geriatrics, Shenzhen People’s Hospital (The Second Clinical Medical College, Jinan University; The First Affiliated Hospital, Southern University of Science and Technology), Shenzhen, 518020 Guangdong China; 3 The Biobank of National Innovation Center for Advanced Medical Devices, Shenzhen People’s Hospital, Southern University of Science and Technology, Shenzhen, China

**Keywords:** Ubiquitylation, Chronic kidney disease

## Abstract

Transforming growth factor-β (TGF-β) is the primary factor that drives fibrosis in most, if not all, forms of chronic kidney disease. In kidneys that are obstructed, specific deletion of *Sirt2* in renal tubule epithelial cells (TEC) has been shown to aggravate renal fibrosis, while renal tubule specific overexpression of *Sirt2* has been shown to ameliorate renal fibrosis. Similarly, specific deletion of *Sirt2* in hepatocyte aggravated CCl4-induced hepatic fibrosis. In addition, we have demonstrated that SIRT2 overexpression and knockdown restrain and enhance TGF-β-induced fibrotic gene expression, respectively, in TEC. Mechanistically, SIRT2 reduced the phosphorylation, acetylation, and nuclear localization levels of SMAD2 and SMAD3, leading to inhibition of the TGF-β signaling pathway. Further studies have revealed that that SIRT2 was able to directly interact with and deacetylate SMAD2 at lysine 451, promoting its ubiquitination and degradation. Notably, loss of SMAD specific E3 ubiquitin protein ligase 2 abolishes the ubiquitination and degradation of SMAD2 induced by SIRT2 in SMAD2. Regarding SMAD3, we have found that SIRT2 interact with and deacetylates SMAD3 at lysine 341 and 378 only in the presence of TGF-β, thereby reducing its activation. This study provides initial indication of the anti-fibrotic role of SIRT2 in renal tubules and hepatocytes, suggesting its therapeutic potential for fibrosis.

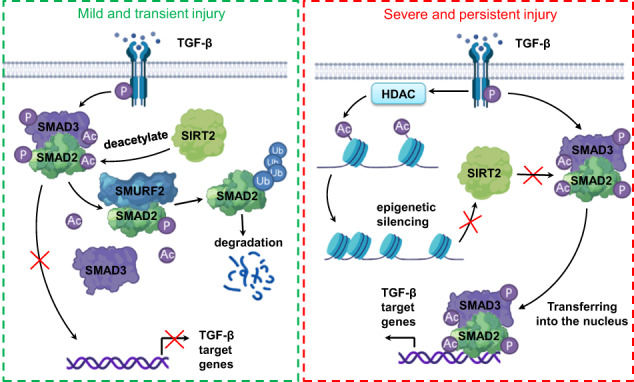

## Introduction

Renal fibrosis is a common pathological feature of chronic kidney disease (CKD) including diabetic kidney disease (DKD) and obstructive nephropathy [1], and its process significantly reduces the regenerative potential of the kidney, leading to a decrease in function [2]. It has been established that renal tubular cells play a central role in the renal fibrotic response following acute injury and CKD [3]. Furthermore, hereditary proximal tubule-specific damage exhibits characteristics of CKD such as renal fibrosis [4]. Tubulointerstitial fibrosis (TIF) and glomerulosclerosis have also been detected in mice with tubular epithelial cell-specific injury [5, 6]. As the principal manifestation of tubular injury, TIF is characterized by the deposition of fibronectin (FN1) and collagen type III (COL3) in the extracellular matrix (ECM) [7, 8]. In DKD, renal tubular epithelial cells (TECs) lose their epithelial characteristics after initiating the expression of fibroblast markers, ultimately leading to ECM remodeling and TIF [9]. However, the molecular mechanisms of TIF remain unclear, and there are limited therapeutics for the effective treatment of renal fibrosis [10].

Transforming growth factor beta (TGF-β) is a well-known key mediator of renal tubular fibrosis (RIF) [11]. TGF-β initiates fibrosis by binding to TGF-β II type receptors (TGFβRII) in renal tubular epithelial cells and activating TGF-β I type receptors, leading to SMAD2/3 phosphorylation [12, 13]. The activated SMAD complex enters the nucleus and transcribes fibrosis-associated proteins, such as collagen type I (COL1A1) and smooth muscle actin (SMA) [14, 15]. SMAD2/3 requires phosphorylation for nuclear translocation and transcriptional regulation of the target genes of fibrosis [16]. Previous research has suggested that acetylation can also modulate the transcriptional activity of SMAD. For example, acetylation of lysine (Lys) 54 is critical for SMAD2 phosphorylation and nuclear translocation [17]. In addition, acetylation of Lys19 on SMAD2 induces a conformational change in the MH1 domain, allowing its DNA binding domain to interact with DNA [18]. Furthermore, coactivators such as p300, CREB Binding Protein (CBP), and P300/CBP-Associated factor (P/CAF) acetylate Lys19 of SMAD2 in a TGF-β-dependent manner [18]. Although acetylation is reversible, the specific deacetylase responsible for SMAD2 deacetylation and its involvement in tubular epithelial cells and the process of renal fibrosis remain to be fully elucidated.

Sirtuin (SIRT) proteins, an evolutionarily conserved family of NAD^+^-dependent protein deacetylases, have been shown to play important roles in the post-translational regulation of many metabolic and cytoprotective processes. In mammals, seven sirtuins (SIRT1-7) associated with protein deacetylation constitute an evolutionarily conserved enzyme family. Among these, SIRT2 is a major metabolic regulator that deacetylates multiple protein targets [19, 20]. For instance, the hepatic overexpression of SIRT2 has been shown to improve oxidative stress, mitochondrial dysfunction and insulin sensitivity in obese mice [21]. In addition, SIRT2 has been demonstrated to deacetylate and inactivate the inflammasome in NLRP3 in macrophages, thereby preventing and potentially reversing age-associated inflammation and insulin resistance [22]. However, the role of the cytosolic SIRT2 in fibrotic kidney disease is not fully understood. Although the expression of SIRT2 has been detected in the renal tubules of disease-free human and mouse kidneys [23], the role of SIRT2 in proximal tubular epithelial cells (TECs) in renal fibrosis has not been documented. This study aimed to investigate the anti-fibrotic effect of SIRT2 induced by unilateral ureteral occlusion (UUO) and unilateral renal ischemia reperfusion injury (uIRI) in a fibrotic mouse model and human biopsy specimens. In addition, we sought to identify a new role for SIRT2 in regulating the TGF-β/SMAD signaling pathway to suppress renal fibrosis.

## Results

### SIRT2 was substantially downregulated in human and mouse fibrotic kidneys

**Fig. 1:**
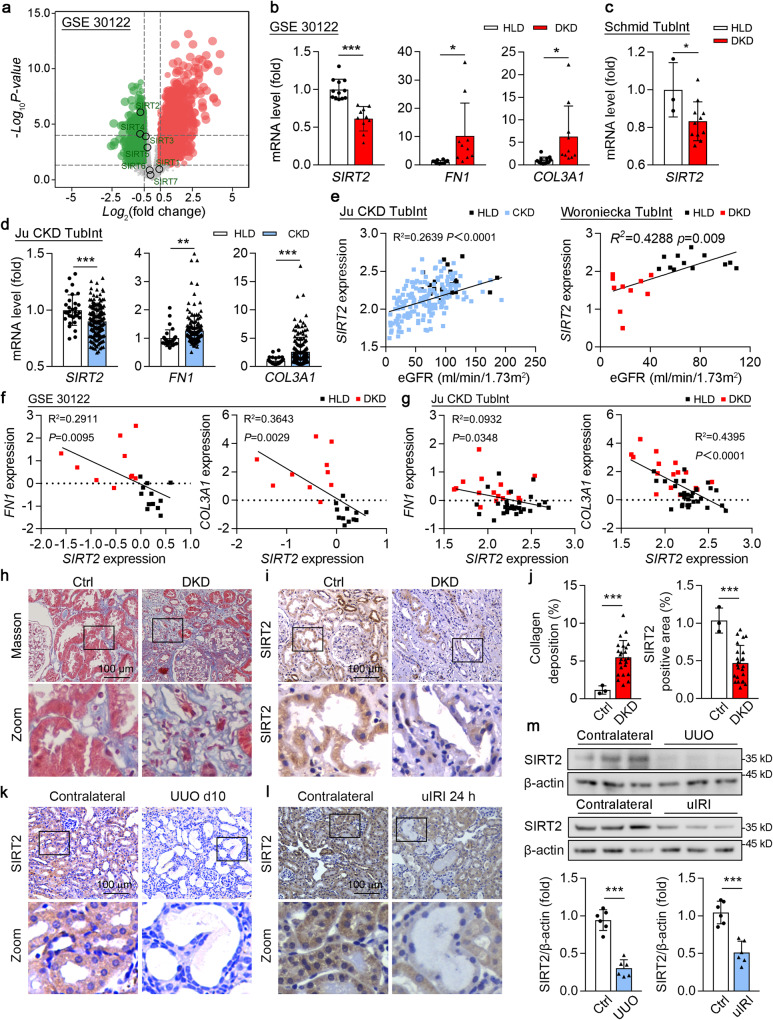
SIRT2 was reduced in the human and mouse fibrotic kidneys. **a**, **b** Reanalysis of the database obtained from GEO database (GSE30122) on human kidney specimens from patients with DKD (healthy control *n* = 11, DKD *n* = 10) showed that *SIRT2* was significantly downregulated, and *FN1* and *COL3A1* were significantly upregulated in the tubulointerstitial (Tublnt) from DKD patients. **c** Reanalysis of the data obtained from the Nephrin database showed that *SIRT2* was significantly downregulated in the Tublnt from DKD patients (healthy control *n* = 3, DKD *n* = 11). **d** Reanalysis of the data obtained from the Nephrin database (Ju CKD Tublnt) showed that *SIRT2* was significantly downregulated, and *FN1* and *COL3A1* were significantly upregulated in the tublnt of kidney tissues from CKD patients (*n* = 170). **e** Spearman correlations were analyzed between *SIRT2* transcription level and eGFR (ml/min/1.73 m^2^) in HLD and patients with CKD (left) or DKD (right), respectively. **f**, **g** Spearman correlation analysis of *SIRT2* and *FN1*, *SIRT2* and *COL3A1* in renal Tublnt of DKD patients. **h–j** Representative images of Masson’s trichrome staining (**h**) and SIRT2 immumohistochemical staining (**i**) in the kidney sections from patients with minimal change disease [control (Ctrl), *n* = 1], and DKD (*n* = 8) (scale bar = 100 μm). **j** Quantitative analysis of Masson’s trichrome and SIRT2 immunofluorescence staining in the indicated human renal biopsies (**f** and **g**), the collagen deposition (left) and SIRT2 positive area (right) quantified in the kidney sections in 3 fields per patients at ×100 magnification. **k–m** Representative images of SIRT2 immumohistochemical staining in mouse fibrotic kidneys induced by UUO (**k**) or uIRI (**l**), compared with kidneys from mice after sham surgery (scale bar = 100 μm). **m** Western blot analysis of SIRT2 and β-actin expression in mouse fibrotic kidneys induced by UUO or uIRI, compared with contralateral kidneys (control). The quantitative results are shown in the bottom panel. β-actin was used as the loading control (*n* = 6). For all panels, data are presented as mean ± SD. *P < 0.05, **P < 0.01, ***P < 0.001 by a one-way ANOVA with a Bonferroni correction test (**b**, **c**, **d**, **j**, **m**). Spearman’s correlation is also shown (**e**, **f**, **g**).

We initially found that *SIRT2* expression was reduced in tubulointerstitial tissue (Fig. 1a, b) but not in the glomerulus (Fig. S1a–c) of renal biopsy specimens obtained from patients with DKD (GSE30122). In addition, markers for kidney fibrosis, such as *COL3A1* and *FN1*, showed significant increased compared to those from healthy donors (Fig. 1b). Consistent with these findings, reanalysis of microarray data obtained from Nephroseq (https://www.nephroseq.org/) revealed a decrease in *SIRT2* expression in tubulointerstitial tissue of renal biopsy specimens from patients with DKD compared to the healthy living donors (HLD) (Fig. 1c). Furthermore, *SIRT2* expression was also significantly decreased in tubulointerstitial tissue from patients with CKD, and markers for kidney fibrosis, such as *COL3A1* and *FN1*, showed significant increase, compared to those from healthy donors (Fig. 1d). Further analysis showed that *SIRT2* expression was positively correlated with the estimated glomerular filtration rate (eGFR) in HLD and in patients with CKD or DKD (Fig. 1e). Moreover, *SIRT2* transcription levels were negatively correlated with *FN1* and *COL3A1* in the tubulointerstitium of kidney tissues from patients with DKD (Fig. 1f, g). Immunohistochemistry also studies revealed a marked decrease in the intensity of SIRT2 staining in kidney sections from patients with DKD compared to those of the control group (patients diagnosed with minimal change disease) (Fig. 1h–j). Immunohistochemistry (Fig. 1k, l) and immunoblotting (Fig. 1m) also showed substantial downregulation of SIRT2 protein in fibrotic mouse kidney tissues induced by UUO and uIRI (Fig. S2a–d), both of which are characterized by substantial renal fibrosis [24, 25]. SIRT2 showed abundant expression in the renal tubules and moderate expression in the glomeruli (Fig. 1i, k, l). Notably, the decrease in SIRT2 was predominantly observed in the dilated tubules, which are the major site for tubulointerstitial fibrosis (Fig. 1i, k, l). Taken together, the observed downregulation of SIRT2 mRNA and protein levels in fibrotic human and mouse kidneys, along with its negative correlation with fibrogenic ECM protein genes, suggests a potential role for SIRT2 in the pathogenesis of kidney fibrosis.

### Renal tubular epithelial cell-specific deletion of Sirt2 aggravated the extent of kidney fibrosis in UUO mice

**Fig. 2:**
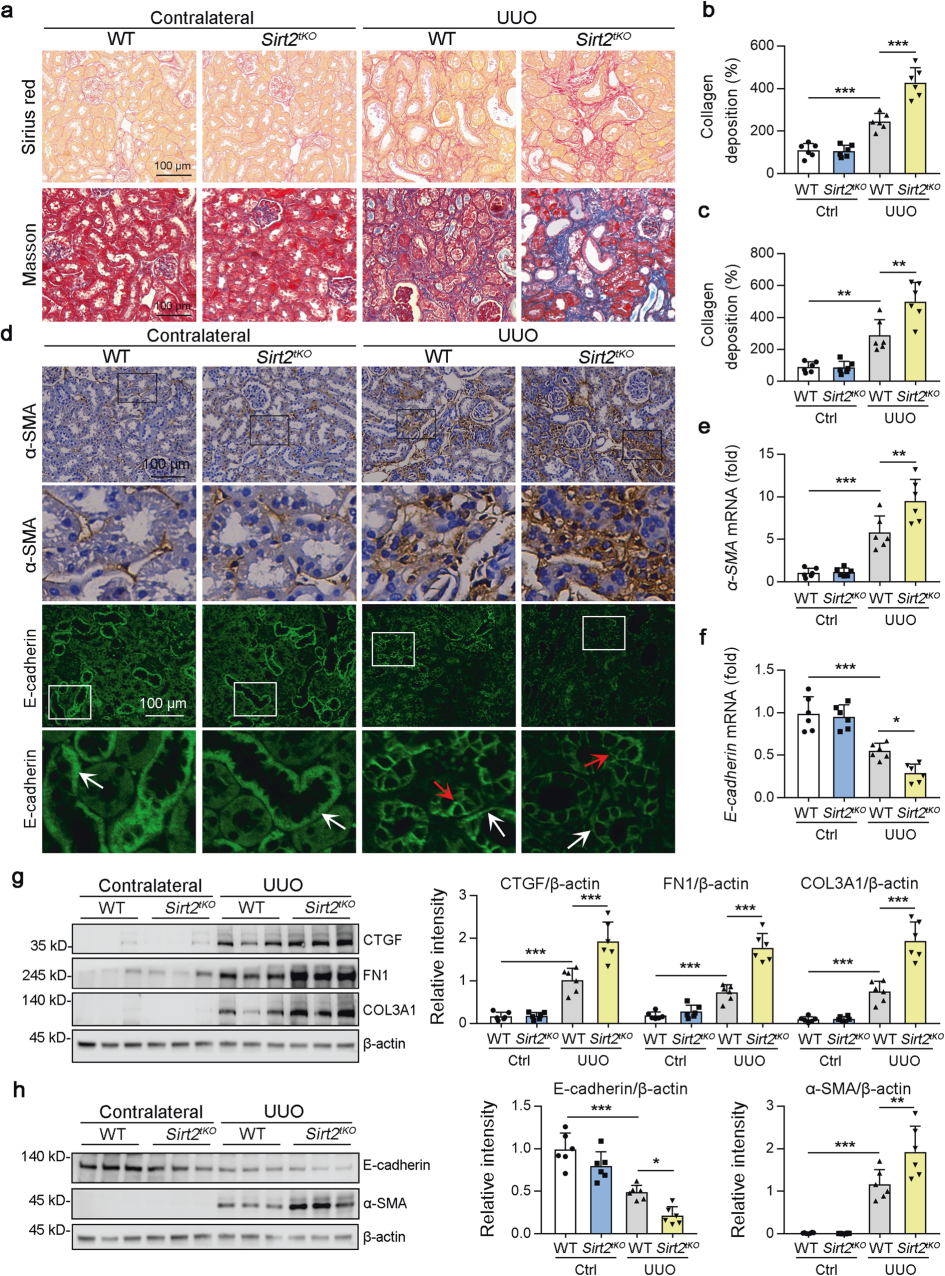
Knockout of SIRT2 in renal tubules aggravated diabetic renal fibrogenesis. Tubule-specific *Sirt2* knockout mice (*Sirt2*^*tKO*^) were generated by crossbreeding *Pax8*-Cre mice with floxed *Sirt2* mice on a C57BL/6 background. **a–c** Representative images of Sirius red and Masson’s trichrome staining in kidney sections from WT and *Sirt2*^*tKO*^ mice at day 7 post-surgery. The collagen deposition of Sirius red staining (**a**, the upper panel) and Masson’s trichrome staining (**a**, the bottom panel) were quantified in the kidney sections in 3 fields per mouse at ×100 magnification (**b** is for Sirius red staining, **c** is for Masson’s trichrome staining [*n* = 6]). **d–f** Representative images of SIRT2 immunohistochemical staining and E-cadherin immunofluorescence staining are shown in panel d, and the mRNA level of *α-SMA* (**e**) and *E-cadherin* (**f**) were determined by qPCR in the kidney samples from mice (*n* = 6). The boxed areas in the upper panels are enlarged in the lower panels. The polarized distribution of E-cadherin in the basolateral membrane of tubules is indicated by white arrows; re-distribution of E-cadherin to the apical membrane of tubules is represented by red arrows. **g**, **h** Western blot analysis of CTGF, FN1, COL3A1, E-cadherin, α-SMA, and β-actin in the kidney samples from WT and *Sirt2*^*tKO*^ mice at day 7 post-surgery along with quantitative results shown in the right panel. β-actin was used as the loading control (*n* = 6). For all panels, data are presented as mean ± SD. *P < 0.05, **P < 0.01, ***P < 0.001 by one-way ANOVA with a Bonferroni correction test.

In the previous section, we reported that SIRT2 was predominantly distributed in renal tubules and that SIRT2 conditional knockout mice specific to TECs were generated (*Sirt2*^*tKO*^; *Pax8*-cre×*Sirt2*^*fl/fl*^). To determine the effects of SIRT2 on the development of kidney fibrosis in vivo, wild type (WT) and *Sirt2*^*tKO*^ mice were subjected to UUO (Fig. S3a,b). First, we found that the kidneys of *Sirt2*^*tKO*^ mice displayed a similar kidney structure and ratio of kidney weight to body weight, urinary albumin excretion rate, albumin to creatinine ratio, and 24 h urine volume compared to WT mice (Fig. S4 a–g). Sirius Red and Masson’s trichrome staining of kidney sections showed extensive renal fibrosis in WT mice following UUO (Fig. 2a–c). Consistent results showed that the levels of CTGF, FN1, COL3A1, and α-SMA, which are markers of kidney fibrosis, were upregulated in WT mice following UUO (Fig. 2d–h). At day 7 post-UUO surgery, the extent of renal fibrosis was induced in *Sirt2*^*tKO*^ kidneys (Fig. 2a–c); they displayed higher levels of CTGF, FN1, COL3A1, and α-SMA compared to WT mice after UUO surgery (Fig. 2d–h). In addition, UUO surgery reduced E-cadherin expression [26] and induced E-cadherin redistribution to the apical membrane in WT mice [27]. *Sirt2*^*tKO*^ aggravated the loss of E-cadherin (Fig. 2h) and promoted E-cadherin redistribution into the apical membrane (Fig. 2d). Above all, the loss of *Sirt2* in renal tubular epithelial cells was shown to augment the extent of kidney fibrosis in UUO mice.

### Renal tubular epithelial cell SIRT2 overexpression alleviated the extent of kidney fibrosis following injury

**Fig. 3:**
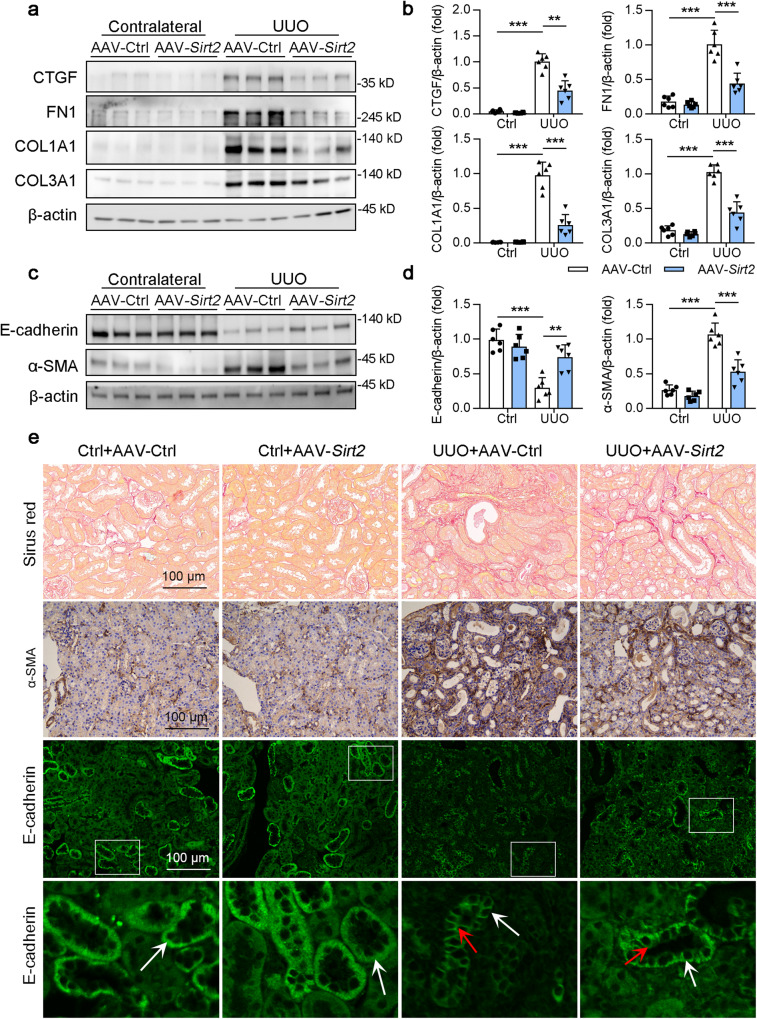
Overexpression of SIRT2 in renal tubular alleviated UUO-induced renal fibrogenesis. AAV-Ctrl or AAV-*Ggt* (gamma-glutamyltransferase 1)-*Sirt2* was injected into bilateral kidneys of mice in situ at five independent points in situ. After 2-week transfection, the mice received UUO surgery, and the contralateral kidneys were used as control. **a–d** Western blot analysis of CTGF, FN1, COL1A1, COL3A1, E-cadherin, α-SMA, and β-actin in the kidneys from mice at day 10 post-surgery, with quantitative results shown in the right panel, and β-actin used as the loading control (*n* = 6). **e** Representative images of Sirius red staining, α-SMA immunohistochemical staining, and E-cadherin immunofluorescence staining were shown in the kidney from mice at day 10 post-surgery. The boxed areas in the upper panels are enlarged in the lower panels. The polarized distribution of E-cadherin in the basolateral membrane of tubules was indicated by white arrows; re-distribution of E-cadherin to the apical membrane of tubules is indicated by red arrows. Key in (**b**) also applies to (**d**). For all panels, the data are presented as mean ± SD. *P < 0.05, **P < 0.01, ***P < 0.001 by one-way ANOVA with a Bonferroni correction test.

Next, opposing phenotypes were examined in AAV9-*Ggt*-*Sirt2*-mediated, renal tubular epithelial cell-specific SIRT2 overexpressed mice (Fig. S3c–f). SIRT2 overexpression in UUO mice reduced the protein levels of COL1A1, COL3A1, CTGF, FN1 (Fig. 3a, b), and α-SMA (Fig. 3c, d). This also caused reduced collagen deposition (assessed by Sirius red staining; Fig. 3e), retarded E-cadherin loss (Fig. 3c, d), and redistribution to the apical membrane (Fig. 3e). *Sirt2* overexpression also resulted in significant reductions of collagen deposition (assessed by Sirius red staining and Masson’s trichrome staining; Fig. S5a, b), and the expression of COL1A1, COL3A1, CTGF, FN1, and α-SMA in the kidneys of uIRI mice (Fig. S5c–e). This was consistent with the observations in the uIRI model. Consistently, *Sirt2* overexpression inhibited E-cadherin loss and redistribution to the apical membrane in the kidneys of uIRI mice (Fig. S5f). Taken together, these results suggest that SIRT2 overexpression in renal tubular epithelial cells alleviates the development of renal fibrosis in UUO and uIRI mice.

### SIRT2 repressed the TGF-β signaling pathway

**Fig. 4:**
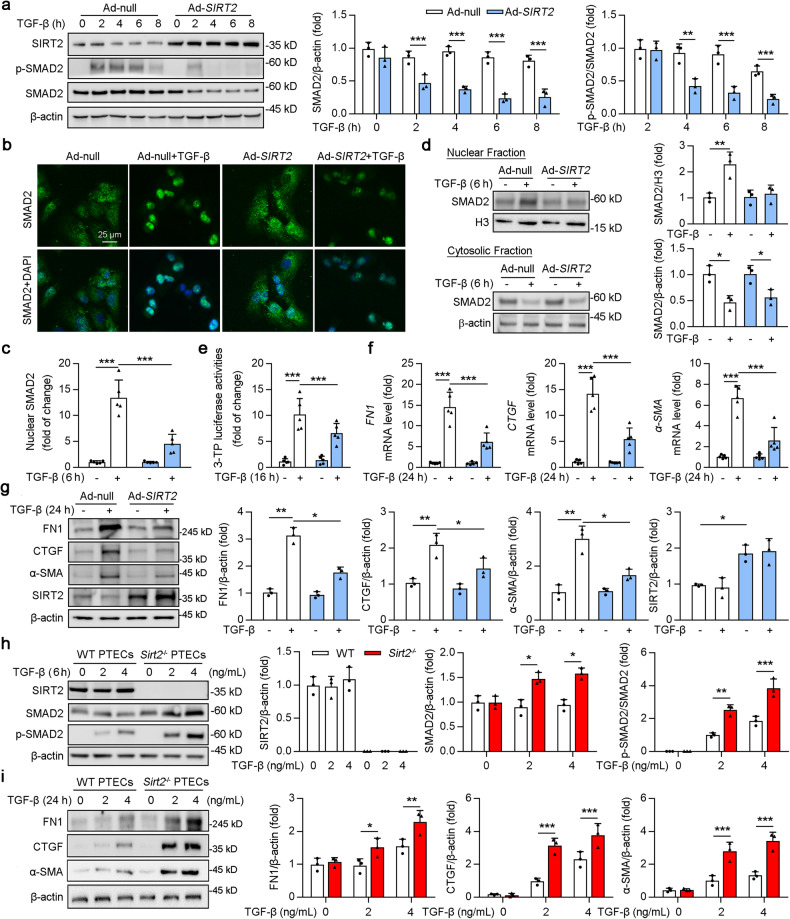
SIRT2 inhibited TGF-β/SMAD signaling in renal tubule epithelial cells. **a** HK2 cells were transfected with Ad-null or Ad-*SIRT2* for 24 h, and treated with or without 2 ng/ml TGF-β for indicated times. Western blot analysis (left) of SIRT2, phosphorylation of SMAD2 (p-SMAD2), SMAD2, and β-actin, and the quantitative results are shown in the right panel (*n* = 3). **b–d** HK2 cells were transfected with Ad-null or Ad-*Sirt2* for 24 h, and treated with 6 ng/ml TGF-β for 6 h. Representative images of SMAD2 immunofluorescence staining are shown in (**b**), and densitometry quantification of nuclear levels of SMAD2 are shown in (**c**). **d** Western blot analyses (left) of SMAD2 in the fractions extracted from HK2 cells, and the quantitative results are shown in the right panel (*n* = 3). **e** 3TP-Lux luciferase activity assay in HEK293T cells after transfection of the 3TP-Lux plasmid, a renilla plasmid, and Ad-null or Ad-*SIRT2* for 24 h, followed by the treatment with or without 2 ng/ml TGF-β for 16 h. Relative luciferase activity is presented as folds of that in the cells with transfection of Ad-null (*n* = 5). **f**, **g** HK2 cells were transfected with Ad-null or Ad-*SIRT2* for 24 h, and treated with or without 2 ng/ml TGF-β for 24 h. **f** The mRNA level of *FN1, CTGF*, and *α-SMA* as determined by qPCR (*n* = 5). **g** Western blot analysis of FN1, CTGF, α-SMA, SIRT2, and β-actin in HK2 cells, with quantitative results shown in the right panel (*n* = 3). **h** PTECs isolated from *Sirt2* knockout (*Sirt2*^−/−^) or wild type (WT) mice were treated with 2 or 4 ng/ml TGF-β for 6 h. Western blot analysis of SIRT2, SMAD2, p-SMAD2, and β-actin, and the quantitative results are shown in the right panel (*n* = 3). **i** PTECs isolated from *Sirt2* knockout (*Sirt2*^−/−^) or wild type (WT) mice were treated with 2 or 4 ng/ml TGF-β for 24 h. Western blot analysis of FN1, CTGF, α-SMA, SIRT2, and β-actin in HK2 cells, with quantitative results shown in the right panel (*n* = 3). The key in (**a**) also applies to (**d**–**g**); the key in (**h**) also applies to (**i**). For all panels, data are presented as mean ± SD. *P < 0.05, **P < 0.01, ***P < 0.001 by a one-way ANOVA with a Bonferroni correction test.

To fully elucidate the molecular pathways behind the positive effects of SIRT2 on renal fibrosis, SIRT2-interacting proteins were screened for using a bioinformatics tool. A potential interaction between SIRT2 and the top 20 genes associated with renal fibrosis was generated by the STRING database, a predictive web interface for gene functions (Fig. S6a). Among these target proteins (Fig. S6a), SMAD2 and SMAD3 showed the strongest correlation with SIRT2. SMAD proteins are key mediators of canonical TGF-β signaling, which transduces TGF-β signals from the cell surface into the nucleus to regulate transcription [28]. Next, we performed online protein-protein docking via GRAMM-X (http://vakser.compbio.ku.edu) [29], and found that the △G (Kcal/mol) of SIRT2 and SMAD2 and SMAD3 were −16.6 and −7, respectively (Fig. S6b). Hence, we firstly investigated whether SIRT2 regulates the expression or activity of SMAD2 in the HK2 cells (human tubular epithelial cell line). However, SIRT2 overexpression did not significantly change the protein levels of SMAD2 in the absence of TGF-β stimulation (Fig. 4a, 0 h TGF-β treatment). Interestingly, we found that the phosphorylation and protein levels of SMAD2 decreased in SIRT2-overexpressing cells after 2 h of TGF-β treatment compared to those in control group. Previous studies have shown that TGF-β-mediated activation of SMAD2 leads to multi-ubiquitination and subsequent degradation of SMAD2 by the proteasome [30–32]. Intriguingly, treatment of HK2 cells with TGF-β and cycloheximide (CHX), which eliminates translational regulation of protein expression, gradually induced the degradation of SMAD2 under TGF-β stimulation for 8–14 h (Fig. S6c). Next, a time-course experiment showed that SMAD2 levels were not obviously reduced under TGF-β stimulation for 0–14 h in HK2 cells (Fig. S6d). This might be a result of an increase in SMAD2 transcription in response to TGF-β stimulation [33, 34], which compensates for the degradation of SMAD2 in a feedback manner. These results suggest that SIRT2 overexpression break the expression balance of SMAD2 under TGF-β stimulation.

To investigate the nuclear translocation of SMAD2, we performed immunofluorescence analysis, and found that TGF-β stimulation promoted the nuclear localization of SMAD2. However, this nuclear translocation was inhibited by SIRT2 overexpression (Fig. 4b, c, column 2 vs 4). Consistent with this, the measurement of nuclear fractions further demonstrated that TGF-β-induced nuclear translocation of SMAD2 was restrained after SIRT2 overexpression (Fig. 4d, column 2 vs 4). Furthermore, SIRT2 overexpression suppressed the transcriptional activity of SMAD2 induced by TGF-β, as shown by the 3TP-Lux luciferase assay [35] (Fig. 4e, column 2 vs 4). In addition, SIRT2 overexpression inhibited TGF-β-induced transcription of *FN1*, *CTGF*, and *α-SMA* (Fig. 4f, column 2 vs 4). We also measured the protein levels of TGF-β target genes (such as FN1, CTGF, and α-SMA). Consistently, TGF-β-induced upregulation of FN1, CTGF, and α-SMA protein levels was attenuated by SIRT2 overexpression (Fig. 4g). In agreement, we found a similar protein level of SMAD2 between PTECs isolated from WT or *Sirt2*^*−/*−^ mice without TGF-β treatment (Fig. 4j). Under TGF-β stimulation, *Sirt2*^*−/*−^ PTECs showed increased phosphorylation and protein levels of SMAD2 compared to those in WT PTECs (Fig. 4h), and TGF-β-induced upregulation of FN1, CTGF, and α-SMA protein levels was enhanced by SIRT2 knockout (Fig. 4i). Thus, we demonstrated that SIRT2 inhibits TGF-β/SMAD signaling in renal tubule epithelial cells.

### SIRT2 deacetylated Smad2 and promotes its degradation

**Fig. 5:**
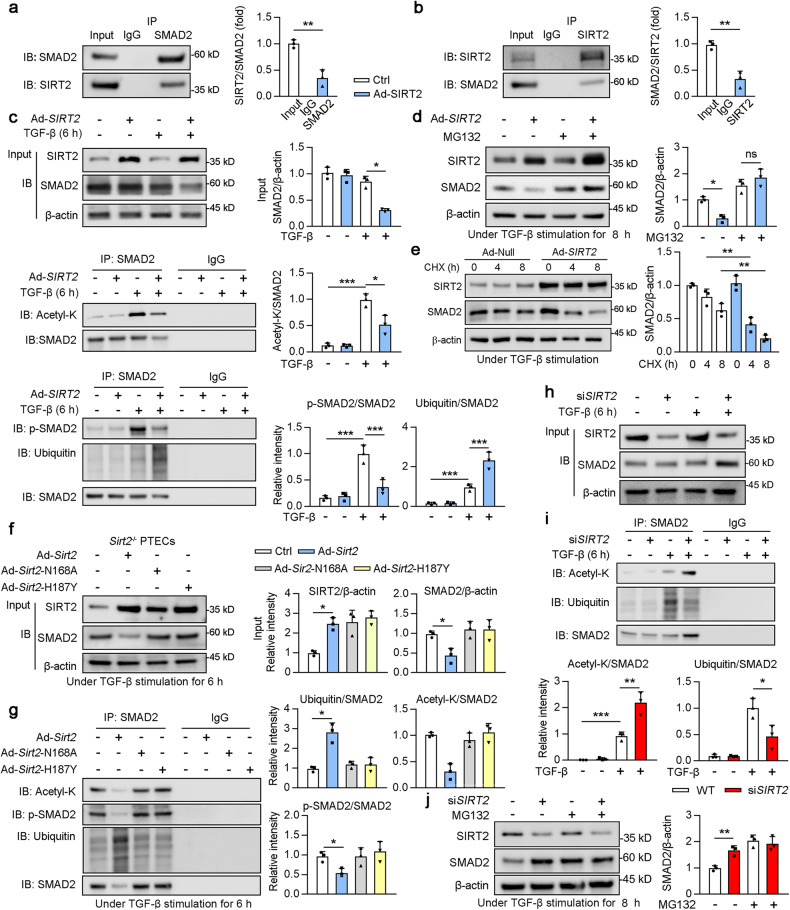
SIRT2 interacts with SMAD2 and promoted its degradation. **a**, **b** HK2 cell lysates were subjected to co-immunoprecipitation (Co-IP) with anti-SIRT2 or anti-SMAD2 antibody, respectively. Normal rabbit IgG was used as the control, and western blotting was conducted using the indicated antibodies. **c** HK2 cells transfected with Ad-Null or Ad-*SIRT2* for 24 h, and treated with or without 2 ng/ml TGF-β for 6 h. HK2 cell lysates were subjected to Co-IP with anti-SMAD2 antibody, followed by western blotting using the indicated antibodies. The quantitative results are shown in the right panel (*n* = 3). **d** HK2 cells transfected with Ad-Null or Ad-*SIRT2* for 24 h, and treated with or without protease inhibitor MG132 (20 μM) in the present of TGF-β for 8 h. Western blot analysis of SIRT2, SMAD2, and β-actin in HK2 cells, with the quantitative results shown in the right panel (*n* = 3). **e** HK2 cells were transfected with Ad-Null or Ad-*SIRT2*, and treated with CHX under TGF-β stimulation for indicated times. Western blot analysis of SIRT2, SMAD2, and β-actin in HK2 cells, with the quantitative results shown in the right panel (*n* = 3). **f**, **g** PTECs isolated from *Sirt2*^−/−^ mice and were transfected with Ad-*Sirt2*, Ad-*Sirt2*-N168A, and Ad-*Sirt2*-H187Y for 24 h, followed by 6 h TGF-β stimulation. PTECs cell lysates subjected to Co-IP with anti-SMAD2 antibody, followed by western blotting using indicated antibodies, and the quantitative results were shown in the right panel (*n* = 3). **h**,**i** *SIRT2* siRNA (si*SIRT2*) or Ctrl siRNA transfected with HK2 cells for 24 h, followed by the treatment with/without 2 ng/ml TGF-β for 6 h. HK2 cell lysates were subjected to Co-IP with anti-SMAD2 antibody, followed by western blotting using indicated antibodies, and the quantitative results were shown in the bottom panel (*n* = 3). **j** HK2 cells transfected with Ctrl siRNA or si*SIRT2* for 24 h, followed by treated with or without protease inhibitor MG132 (20 μM) in the present of TGF-β for 8 h. Western blot analysis of SIRT2, SMAD2, and β-actin in HK2 cells, and the quantitative results were shown in the right panel (*n* = 3). The key in (**a**) also applies to (**b**–**f**); the key in (**g**) also applies to (**h**). For all panels, data are presented as mean ± SD. *P < 0.05, **P < 0.01, ***P < 0.001 by a one-way ANOVA with a Bonferroni correction test.

To understand the molecular mechanism underlying the regulation of SIRT2 on SMAD2, SIRT2 or SMAD2 was immunoprecipitated from cultured HK2 cells, and its binding between SIRT2 and SMAD2 was tested by western blotting. These results suggested that SIRT2 physically interacts with SMAD2 (Fig. 5a, b). Next, we evaluated SMAD2 acetylation status in HK2 cells under TGF-β stimulation and found that TGF-β stimulation enhanced the acetylation of SMAD2, which was decreased by SIRT2 overexpression (Fig. 5c). As an essential post-translational modification, deacetylation is critical for protein stability [36]. Thus, we proposed that SIRT2 deacetylates SMAD2 and promotes its degradation. To test this hypothesis, adenovirus constructs including SIRT2 (Ad-*SIRT2*)- or empty vector (Ad-Null)-transfected HK2 cells were treated with or without protease inhibitor MG132 (20 μM) in the presence of TGF-β for 8 h. SIRT2 overexpression promoted protein destabilization of SMAD2 only in the absence of MG132 (Fig. 5d). Next, SMAD2 was immunoprecipitated from cultured HK2 cells and the phosphorylation and ubiquitylation of SMAD2 were tested by western blotting. SIRT2 overexpression inhibited the phosphorylation of SMAD2 and promoted the ubiquitylation of SMAD2 in the presence of TGF-β (Fig. 5c). To explore the effect of SIRT2 on SMAD2 in more detail, Ad-*SIRT2-* or Ad-Null-transfected HK2 cells were treated with cycloheximide (CHX, a protein synthesis inhibitor) under TGF-β stimulation. SIRT2 overexpression reduced the half-life of SMAD2 (Fig. 5e). To determine whether SIRT2 regulates the function of SMAD2 through its deacetylase activity, we examined SMAD2 acetylation status in *Sirt2*^*−/*−^ PTECs transfected with WT or catalytic mutants of SIRT2 (SIRT2-H187Y [37] or SIRT2-N168A [38]) under TGF-β stimulation. Western blotting analysis indicated that WT, but not catalytic mutant-SIRT2, markedly decreased the protein and acetylation levels of SMAD2 (Fig. 5f, g), indicating that the deacetylase activity of SIRT2 is required for SMAD2 protein-level regulation. Consistently, only WT SIRT2 enhanced the ubiquitination of SMAD2 (Fig. 5g). In agreement with these findings, loss of SIRT2 increased the acetylation of SMAD2, decreased the ubiquitylation of SMAD2, and enhanced its protein stability (Fig. 5h–j). Above all, these results suggest that SIRT2 deacetylated SMAD2 and promoted its ubiquitination and degradation in HK2 cells.

### SIRT2 deacetylated SMAD2 on Lys451

**Fig. 6:**
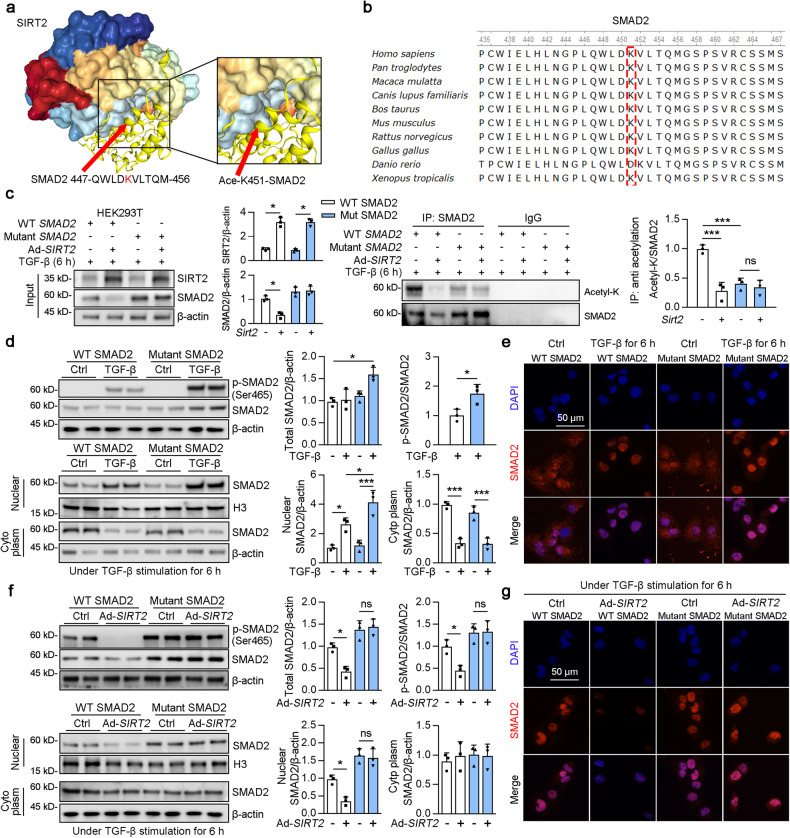
SIRT2 regulated TGF-β signaling by deacetylating lysine 451 on SMAD2. **a** SIRT2-SMAD2 K451 docking with the HDOCK server. High magnification of boxed areas is presented on the right. Left arrow indicates the SMAD2 protein 447-456 peptide; right arrow indicates SMAD2 protein K451 site. **b** The conservation of SMAD2 lysine 451 in different species. **c**, **d** HEK293T cells transfected with Ad-WT *SMAD2*, Ad-mutant *SMAD2*, or Ad*-SIRT2* for 24 h, followed by the treatment of 2 ng/ml TGF-β for 6 h. HEK293T cell lysates were subjected to Co-IP with anti-SMAD2 antibody, followed by western blotting using indicated antibodies, and the quantitative results are shown in the right panel (*n* = 3). **d** HEK293T cells were transfected with Ad-WT *SMAD2* or Ad-mutant *SMAD2* for 24 h, followed by the treatment of with or without 2 ng/ml TGF-β for 6 h. Western blot analyses of p-SMAD2 (Ser465), SMAD2, and β-actin in the whole cells lysates or fractions extracted from HEK293T cells, with quantitative results shown in the right panel (*n* = 3). **e** HEK293T cells transfected with Ad-WT *SMAD2*, Ad-mutant *SMAD2* for 24 h, followed by the treatment with or without 2 ng/ml TGF-β for 2 h. Representative images of SIRT2 immunofluorescence staining are shown in the HEK293T cells (scale bar = 50 μm). **f**, **g** HEK239T cells were transfected with Ad-WT *SMAD2*, Ad-mutant *SMAD2*, or Ad-*SIRT2* as indicated for 24 h, followed by the treatment with 2 ng/ml TGF-β for 6 h. **g** Western blot analyses of p-SMAD2 (Ser465), SMAD2, and β-actin in the whole cells lysates or fractions extracted from HEK293T cells, and the quantitative results are shown in the right panel (*n* = 3). **h** Representative images of SIRT2 immunofluorescence staining are shown in the HEK293T cells (scale bar = 50 μm). Key in (**c**) also applies to (**d**–**g**). For all panels, data are presented as mean ± SD. *P < 0.05, **P < 0.01, ***P < 0.001 by one-way ANOVA with a Bonferroni correction test.

To identify the deacetylation sites of SMAD2 that mediate its interaction with SIRT2, we performed online protein-protein docking using HDOCK (http://hdock.phys.hust.edu.cn/). The server provided docking information about the SIRT2-SMAD2 protein peptide (Table S1), helping to elucidate SIRT2 and SMAD2-Lys451 interactions (Fig. 6a). Lysine 451 in SMAD2 was highly conserved among the different species (Fig. 6b), indicating an important role for Lys451 in SMAD2. WT SMAD2, mutant SMAD2 [the mutation of lysine (K) to arginine (R) at the 451 site], and Ad-*SIRT2* transfected with HEK293T cells as indicated (Fig. 6c). SMAD2 was immunoprecipitated from cultured HE293T cells, and the binding between acetylation and SMAD2 was tested by western blotting (Fig. 6c). As expected, mutant SMAD2 decreased the acetylation of SMAD2 compared to that of WT SMAD2 under TGF-β stimulation (column 3 vs 1), and this reduction could not be further reduced by SIRT2 overexpression (column 4 vs 3). Notably, the Lys451 mutation increased the protein level of SMAD2, suggesting that Lys451 could also be a site for ubiquitination. Interestingly, Lys451 mutation increased the protein level, phosphorylation, and nuclear accumulation of SMAD2 compared to those in WT *SMAD2* transfected cells after 6 h of TGF-β stimulation (Fig. 6d, column 4 vs 2). Next, we performed immunofluorescence staining and found that nuclear accumulation of SMAD2 was enhanced when Lys451 was mutated upon TGF-β stimulation (Fig. 6e). Further experiments showed that SIRT2 overexpression reduced the phosphorylation, protein levels, and nuclear accumulation of WT SMAD2 in HEK293T cells after 2 h of TGF-β stimulation (Fig. 6f, column 2 vs 1), whereas SIRT2 overexpression did not affect these changes in mutant SMAD2 transfected cells (Fig. 6f, column 4 vs 3). Similar results were observed in SMAD2 immunofluorescence staining; SIRT2 overexpression inhibited the nuclear accumulation of SMAD2 in WT SMAD2 transfected HEK293T cells, but not in mutant SMAD2 transfected cells (Fig. 6g). Previous studies have reported that Lys19 [39], Lys 39 [39], Lys 54 [17], and Lys 420 [40] are acetylation sites of SMAD2. Hence, we generated five deacetylated mimetic mutants by mutating lysine to arginine at Lys19, Lys 39, Lys 54, Lys 420, and Lys451. HEK293T cells were transfected with siRNA Ctrl (siCtrl) or siRNA *SIRT2* (si*SIRT2*) and WT *SMAD2* or different mutants (*SMAD2*-K19R, *SMAD2*-K39R, *SMAD2*-K54R, *SMAD2*-K420R, and *SMAD2*-K451R), followed by immunoprecipitation using a SIRT2 antibody and western blotting with an anti-acetylated lysine antibody (Fig. S7). However, the acetylation levels of four mutants (*SMAD2*-K19R, *SMAD2*-K39R, *SMAD2*-K54R, and *SMAD2*-K420R) significantly increased in si*SIRT2* transfected cells, but not *SMAD2*-K451R. These findings suggest that SMAD2 can be acetylated at Lys 451 by SIRT2.

### Mutant of lys451 on SMAD2-inhibited SMURF2 induced ubiquitination

**Fig. 7:**
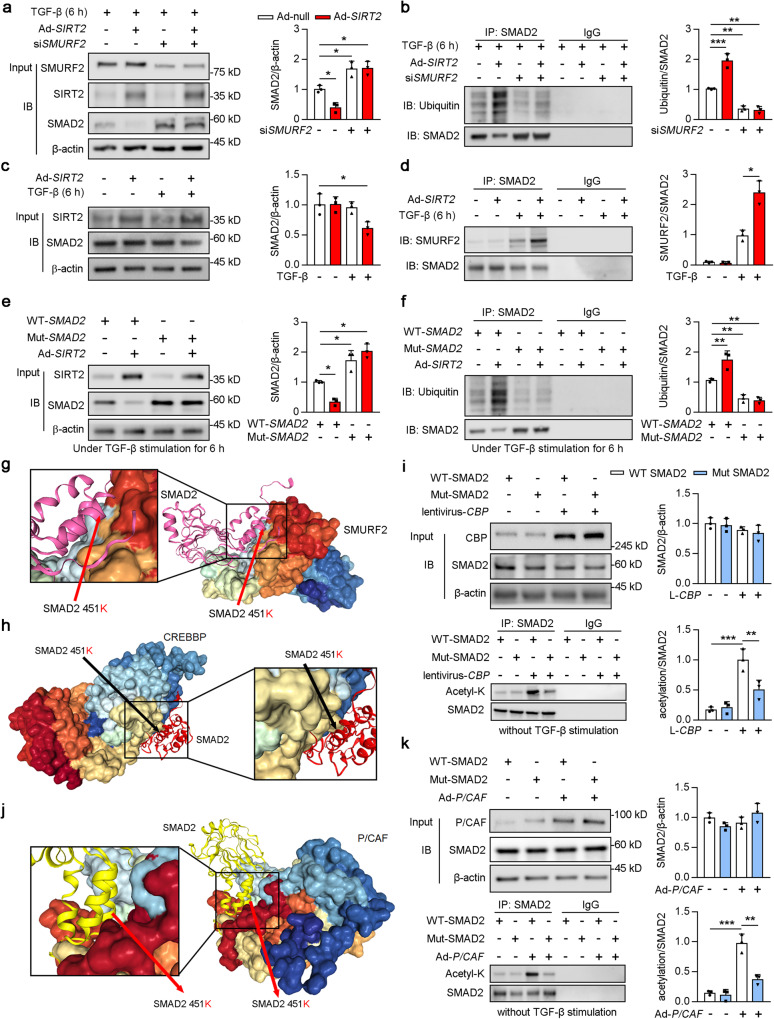
Lysine 451 mutant SMAD2 decreased the ubiquitization induced by SMURF2 and the acetylation of SMAD2 induced by CBP and P/CAF. **a**, **b** HK2 cells transfected with Ad-*SIRT2* and/or si*SMURF2* for 24 h (Ad-Null and siCtrl were used as controls, respectively), and treated with 2 ng/ml TGF-β for 6 h. HK2 cell lysates were subjected to Co-IP with anti-SMAD2 antibody, followed by western blotting using indicated antibodies (**a**), with the quantitative results shown in **b** (*n* = 3). **c**,**d** HK2 cells were transfected with Ad-Null or Ad-*SIRT2* for 24 h, and treated with or without 2 ng/ml TGF-β for 6 h. HK2 cell lysates subjected to Co-IP with anti-SMAD2 antibody, followed by western blotting using indicated antibodies (**c**), with quantitative results shown in the (**d**) (*n* = 3). **e**, **f** HEK293T cells were transfected with Ad-WT *SMAD2*, Ad-mutant *SMAD2*, or Ad-*SIRT2* as indicated for 24 h, and treated with 2 ng/ml TGF-β for 6 h. HEK293T cell lysates subjected to Co-IP with anti-SMAD2 antibody, followed by western blotting using indicated antibodies (**f**). Quantitative results are shown in the right panel (*n* = 3). **g** SMURF2-SMAD2 K451 docking with the HDOCK server. High magnification of boxed areas is presented on the left. Arrow indicates SMAD2 protein K451 site. **h** CBP-SMAD2 K451 docking with the HDOCK server. High magnification of boxed areas is presented on the right. Arrow indicates SMAD2 protein K451 site. **i** HEK239T cells were transfected with lentivirus-*CBP or* lentivirus empty vector for 48 h, and subjected to Ad-WT-*SMAD2* or Ad-Mut-*SMAD2* transfection as indicated for 24 h. HEK293T cell lysates subjected to Co-IP with anti-SMAD2 antibody, and western blotting using indicated antibodies. Quantitative results are shown in the right panel (*n* = 3). **j** P/CAF-SMAD2 K451 docking with the HDOCK server. High magnification of boxed areas is presented on the left. Arrow indicates SMAD2 protein K451 site. **k** HEK239T cells were transfected with Ad-WT-*SMAD2*, Ad-Mut-*SMAD2*, or Ad-*P/CAF* as indicated for 24 h. HEK293T cell lysates subjected to Co-IP with anti-SMAD2 antibody, and western blotting using indicated antibodies. Quantitative results were shown in the right panel (*n* = 3). Key in (**b**) also applies to (**d**, **g**). Key in (**i**) also applies to (**k**). For all panels, data are presented as mean ± SD. *P < 0.05, **P < 0.01, ***P < 0.001 by a one-way ANOVA with a Bonferroni correction test.

SIRT2 overexpression promoted TGF-β-activated SMAD2 degradation (Figs. 4a and 5c). Studies have reported that SMAD ubiquitin regulatory factor 2 (*SMURF2*), an essential negative regulator of TGF-β signalling, interacts with SMAD2 to promote its degradation only in the presence of TGF-β [31]. Hence, we hypothesized that SMURF2 participates in SIRT2-mediated SMAD2 degradation. SIRT2 overexpression decreased the protein level of SMAD2 (column 2 vs 1), which was abolished by si*SMURF2* treatment (Fig. 7a, b). In addition, the role of SIRT2 overexpression in the ubiquitination of SMAD2 (column 2 vs 1) was restrained by si*SMURF2* treatment (Fig. 7a, b). Consistent with a previous study [31], TGF-β stimulation enhanced the interaction between SMAD2 and SMURF2 (Fig. 7c, d; column 3 vs 1). Notably, we found that SIRT2 overexpression further promoted the interaction between SMAD2 and SMURF2 induced by TGF-β (Fig. 7c, d, column 4 vs 3). Hence, HEK293T cells were transfected with Ad-*SIRT2*, Ad-WT*-SMAD2*, or Ad-Mut-*SMAD2* upon TGF-β stimulation, as indicated in Fig. 7e. The Lys451 mutation restrained the ubiquitination of SMAD2 (column 3 vs 1), which did not change after SIRT2 overexpression (Fig. 7f, column 4 vs 3). Interestingly, docking information about SMURF2 and SMAD2 was obtained from HDOCK, suggesting a possible interaction between SMURF2 and SMAD2-Lys451 (Fig. 7g). Furthermore, SMURF2 staining of human kidneys showed strong positivity in tubules [41]. Together, these findings suggested that SIRT2 deacetylated SMAD2 at Lys451 and promoted its ubiquitination.

### Lys451 on SMAD2 was acetylated by CBP and P/CAF

To identify the key factor mediating the acetylation of Lys451 on SMAD2, acetylation prediction tools (GPS-PAIL; http://pail.biocuckoo.org/) were used to help elucidate potential acetylated sites. CBP and KAT2B (also known as P/CAF) were predicted to be the most likely enzymes to acetylate Lys451 on SMAD2 (Table 1). In a previous study, Lys19 on SMAD2 was shown to be acetylated by the coactivators p300, CBP, and P/CAF in a TGF-β-dependent manner [18]. Consistent with this, the results obtained from GPS-PAIL, p300, CBP, and P/CAF were predicted to acetylate Lys19 on SMAD2. Notably, CBP and P/CAF, but not p300, had stronger potential to acetylate Lys451 on SMAD2 compared to Lys19 on SMAD2 (Tables 2 and 3). Notably, we performed online protein-protein docking of SMAD2 and CBP via HDOCK, indicating an interaction between CBP and SMAD2-Lys451 (Fig. 7h). HEK293T cells were transfected with Ad-WT-*SMAD2*, Ad-Mut-*SMAD2*, and adenovirus constructs, including CBP (Fig. 7i). Mutation of Lys451 resulted in decreased acetylation of SMAD2 after CBP overexpression compared to Ad-WT-*SMAD2* transfection (Fig. 7i, column 4 vs 3). Likewise, docking results indicated an interaction between P/CAF and SMAD2-Lys451 (Fig. 7j). Compared to WT-*SMAD2*, Mut-*SMAD2* reduced the acetylation of SMAD2 after P/CAF overexpression (Fig. 7k, column 4 vs 3). Collectively, these findings suggest that CBP and P/CAF may acetylate Lys451 on SMAD2.

**Table 1:** GPS predicts the acetylases of lys451 in SMAD2.

ID	Position	Petide	HAT	Score
Homo SMAD2	451	GPLQWLDKVLTQMGS	CREBBP	0.694
Homo SMAD2	451	GPLQWLDKVLTQMGS	KAT2B	0.404
Homo SMAD2	451	GPLQWLDKVLTQMGS	HAT1	0.133
Homo SMAD2	451	GPLQWLDKVLTQMGS	KAT2A	0.101
Homo SMAD2	451	GPLQWLDKVLTQMGS	EP300	0.006

**Table 2:** GPS predicts lysine sites in SMAD2 may be acetylated by P/CAF.

ID	Position	Petide	HAT	Score
Homo SMAD2	46	KWCEKAVKSLVKKLK	P/CAF	0.817
Homo SMAD2	73	TTQNCNTKCVTIPST	P/CAF	0.679
Homo SMAD2	51	AVKSLVKKLKKTGRL	P/CAF	0.651
Homo SMAD2	63	GRLDELEKAITTQNC	P/CAF	0.606
Homo SMAD2	39	EQNGQEEKWCEKAVK	P/CAF	0.587
Homo SMAD2	43	QEEKWCEKAVKSLVK	P/CAF	0.55
Homo SMAD2	375	WHPATVCKIPPGCNL	P/CAF	0.541
Homo SMAD2	13	PFTPPVVKRLLGWKK	P/CAF	0.477
Homo SMAD2	53	KSLVKKLKKTGRLDE	P/CAF	0.431
Homo SMAD2	54	SLVKKLKKTGRLDEL	P/CAF	0.413
Homo SMAD2	50	KAVKSLVKKLKKTGR	P/CAF	0.404
Homo SMAD2	451	GPLQWLDKVLTQMGS	P/CAF	0.404
Homo SMAD2	121	RLQVSHRKGLPHVIY	P/CAF	0.349
Homo SMAD2	20	KRLLGWKKSAGGSGG	P/CAF	0.339
Homo SMAD2	383	IPPGCNLKIFNNQEF	P/CAF	0.312
Homo SMAD2	19	VKRLLGWKKSAGGSG	P/CAF	0.239
Homo SMAD2	156	CEYAFNLKKDEVCVN	P/CAF	0.239
Homo SMAD2	420	TIRMSFVKGWGAEYR	P/CAF	0.211
Homo SMAD2	157	EYAFNLKKDEVCVNP	P/CAF	0.202
Homo SMAD2	144	LHSHHELKAIENCEY	P/CAF	0.128

**Table 3:** GPS predicts lysine sites in SMAD2 may be acetylated by CREBBP.

ID	Position	Petide	HAT	Score
Homo SMAD2	420	TIRMSFVKGWGAEYR	CREBBP	1.125
Homo SMAD2	50	KAVKSLVKKLKKTGR	CREBBP	0.944
Homo SMAD2	20	KRLLGWKKSAGGSGG	CREBBP	0.911
Homo SMAD2	53	KSLVKKLKKTGRLDE	CREBBP	0.819
Homo SMAD2	46	KWCEKAVKSLVKKLK	CREBBP	0.79
Homo SMAD2	451	GPLQWLDKVLTQMGS	CREBBP	0.694
Homo SMAD2	19	VKRLLGWKKSAGGSG	CREBBP	0.581
Homo SMAD2	43	QEEKWCEKAVKSLVK	CREBBP	0.528
Homo SMAD2	51	AVKSLVKKLKKTGRL	CREBBP	0.44
Homo SMAD2	39	EQNGQEEKWCEKAVK	CREBBP	0.435
Homo SMAD2	54	SLVKKLKKTGRLDEL	CREBBP	0.399
Homo SMAD2	73	TTQNCNTKCVTIPST	CREBBP	0.282
Homo SMAD2	156	CEYAFNLKKDEVCVN	CREBBP	0.274
Homo SMAD2	63	GRLDELEKAITTQNC	CREBBP	0.177
Homo SMAD2	13	PFTPPVVKRLLGWKK	CREBBP	0.157
Homo SMAD2	121	RLQVSHRKGLPHVIY	CREBBP	0.157
Homo SMAD2	157	EYAFNLKKDEVCVNP	CREBBP	0.149
Homo SMAD2	144	LHSHHELKAIENCEY	CREBBP	0.141
Homo SMAD2	375	WHPATVCKIPPGCNL	CREBBP	0.101
Homo SMAD2	383	IPPGCNLKIFNNQEF	CREBBP	0.065

### SIRT2 deacetylated SMAD3 on Lys341 and Lys378

**Fig. 8:**
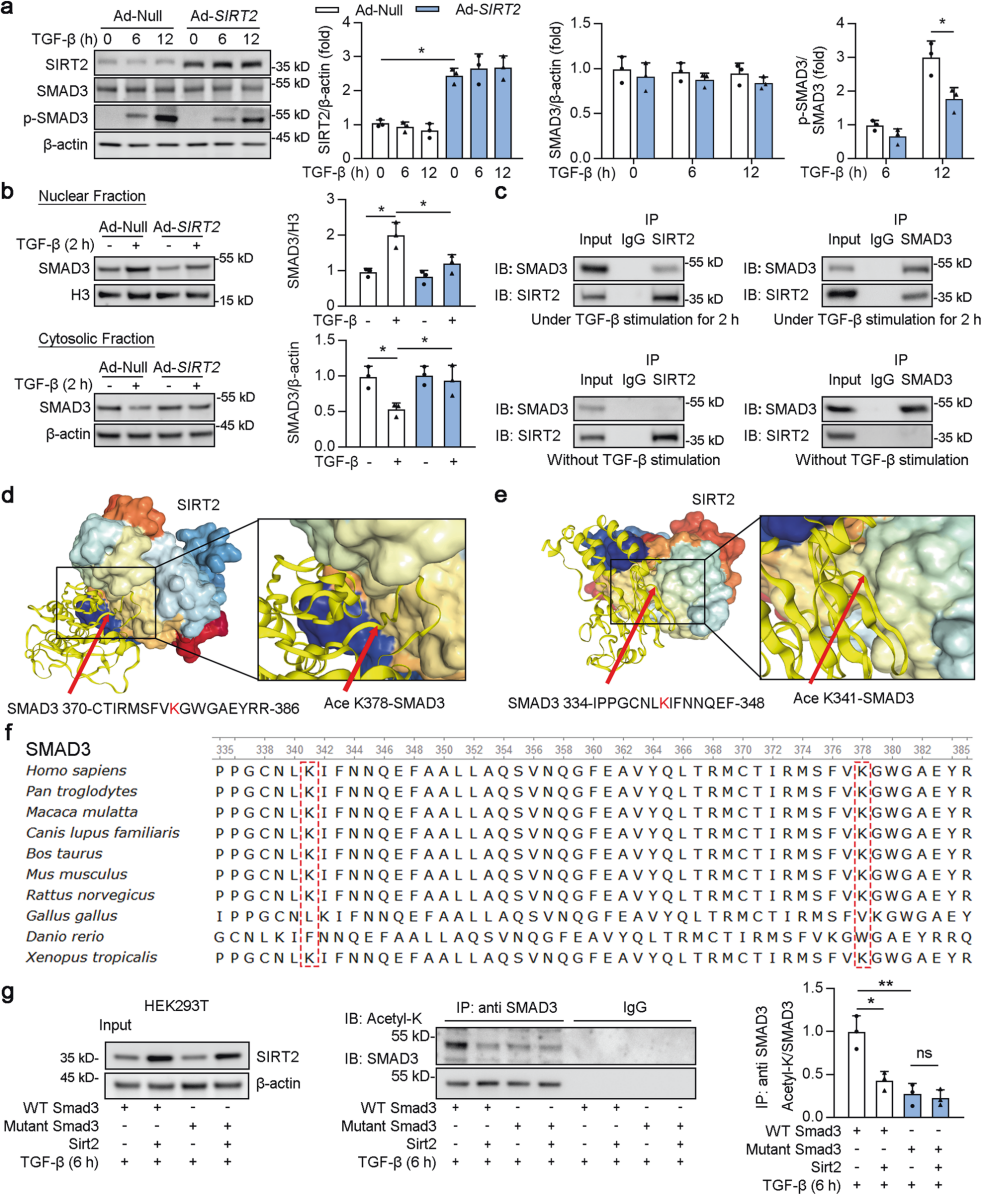
SIRT2 deacetylated Lys341 and Lys378 on SMAD3. **a** HK2 cells were treated with 2 ng/ml TGF-β for indicated times. Western blot analysis of SIRT2, SMAD3, p-SMAD3, and β-actin, and the quantitative results are shown in the right panel (*n* = 3). **b** HK2 cells were transfected with Ad-null or Ad-*Sirt2* for 24 h, and treated with 2 ng/ml TGF-β for 2 h. Western blot analyses (left) of SMAD3 in the fractions extracted from HK2 cells, and the quantitative results are shown in the right panel (*n* = 3). **c** HK2 cell lysates were subjected to Co-IP with anti-SIRT2 or anti-SMAD3 antibody, respectively. Normal rabbit IgG was used as the control, and western blotting was conducted using indicated antibodies. **d**, **e** SIRT2-SMAD2 K341 and K378 docking with the HDOCK server. High magnification of boxed areas is presented on the left. Arrow indicates SMAD3 protein K341 and K378 site. **f** The conservation of SMAD3 lysine 341 and 378 in different species. **g** HEK293T cells were transfected with Ad-WT *SMAD3*, Ad-mutant *SMAD3*, or Ad*-SIRT2* for 24 h, followed by the treatment of 2 ng/ml TGF-β for 6 h. HEK293T cell lysates were subjected to Co-IP with anti-acetylation or anti-SMAD3 antibody, followed by western blotting using indicated antibodies, and the quantitative results are shown in the right panel (*n* = 3). For all panels, data are presented as mean ± SD. *P < 0.05, **P < 0.01, ***P < 0.001 by a one-way ANOVA with a Bonferroni correction test.

Further analysis showed that SIRT2 overexpression decreased the phosphorylation of SMAD3 but not the total protein level of SMAD3 (Fig. 8a). In agreement, SIRT2 overexpression reduced the nuclear localization of SMAD3 induced by TGF-β (Fig. 8b). Next, SIRT2 or SMAD3 was immunoprecipitated from cultured HK2 cells, and the binding between SIRT2 and SMAD3 was tested by western blotting. Intriguingly, these results suggested that SIRT2 physically interacts with SMAD3 only in the presence of TGF-β (Fig. 8c). To identify the deacetylation sites on SMAD3 that mediate its interaction with SIRT2, online protein-protein docking using HDOCK we performed. The server provided docking information on the SIRT2-SMAD3 protein peptide, helping to elucidate SIRT2 and SMAD3-Lys341 and Lys378 interactions (Fig. 8d, e). Lysine 341 and 378 in SMAD3 was highly conserved among different species (Fig. 8f). Previous studies have shown that both the K341R and K378R mutants act as dominant-negative inhibitors in the TGF-β-induced target genes of endogenous TGF-β signal [42]. WT SMAD3, mutant SMAD3 [the mutation of lysine (K) to arginine (R) at the 341 and 378 site], and Ad-*SIRT2* transfected with HEK293T cells as indicated (Fig. 8g). As expected, the mutant SMAD3 decreased the acetylation of SMAD3 compared to that of WT SMAD2 under TGF-β stimulation (column 3 vs 1), and SIRT2 overexpression did not further reduce the acetylation of SMAD3 (column 4 vs 3). Taken together, these findings suggested that SIRT2 deacetylated SMAD3 at Lys341 and Lys378.

### SIRT2 repressed acetylation and nuclear accumulation of SMAD2 and SMAD3 in vivo

**Fig. 9:**
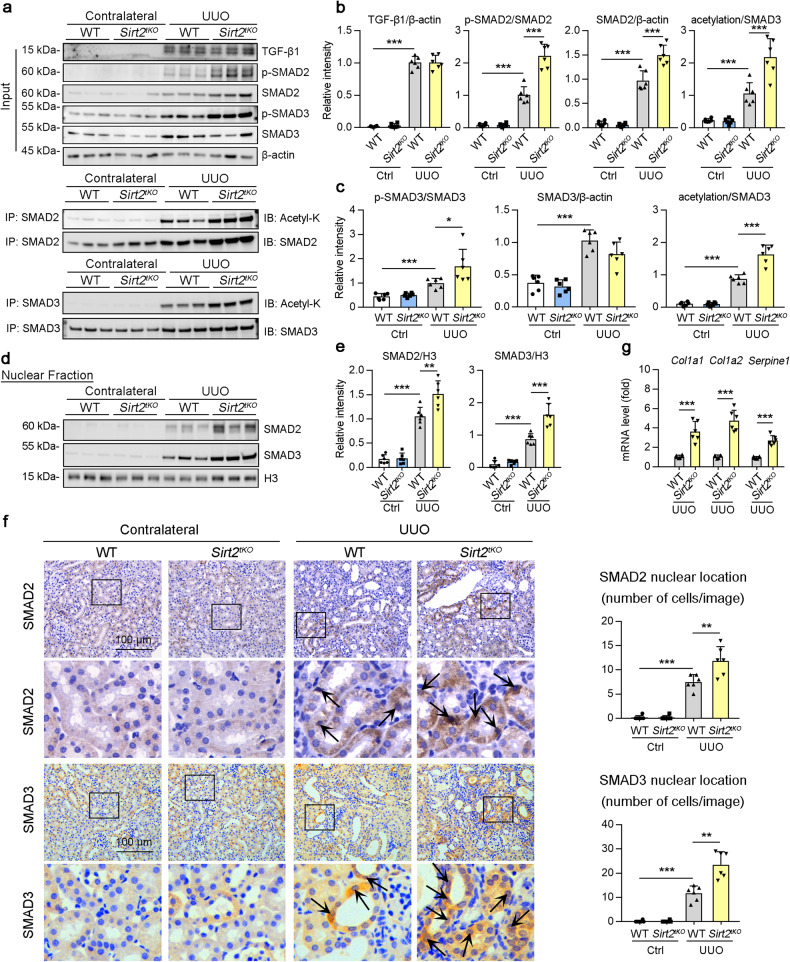
SIRT2 reduced phosphorylation, acetylation and nuclear translocation of SMAD2 and SMAD3 in kidney of UUO mice. **a**, **b** Kidney lysates subjected to Co-IP with anti-SMAD2 or anti-SMAD3 antibody in the WT and *Sirt2*^*tKO*^ mice at day 7 post-surgery, and western blotting using indicated antibodies (**a**). The quantitative results are shown in (**b** and **c**) (*n* = 6). **d**, **e** Western blot analyses (**d**) of nuclear levels of TGF-β1, SMAD2, SMAD3 and H3 in the fractions extracted from the kidney of the WT and *Sirt2*^*tKO*^ mice at day 7 post-surgery. Quantitative results are shown in (**d**) (*n* = 6). **f** Representative images of SMAD2 and SMAD3 immunofluorescence staining in the kidney of WT and *Sirt2*^*tKO*^ mice at day 7 post-surgery, and SMAD2 nuclear location quantified in the kidney sections in 3 fields per mouse at ×100 magnification (*n* = 6). **g** qPCR analysis of the mRNA level of *Col1a1*, *Col1a2*, and *Serpine1* in the kidney of mice (*n* = 6). For all panels, data are presented as mean ± SD. *P < 0.05, **P < 0.01, ***P < 0.001 by a one-way ANOVA with a Bonferroni correction test.

Next, we examined the phosphorylation and acetylation of SMAD2 and SMAD3 in kidney samples from WT or *Sirt2*^*tKO*^ mice on day 7 post-UUO. Consistently, the phosphorylation and acetylation of SMAD2 and SMAD3, but not the protein level of TGF-β1, were significantly higher in *Sirt2*^*tKO*^ UUO kidneys than in WT UUO kidneys (Fig. 9a–c, column 4 vs 3). Furthermore, renal tubule knockout of *Sirt2* promoted the nuclear accumulation of SMAD2 and SMAD3 in the kidneys of mice at day 7 post-UUO compared to that in the kidneys of WT UUO mice (Fig. 9d, e, column 4 vs 3). When compared with WT UUO kidneys, SMAD2 and SMAD3 immunohistochemical staining also showed that the nuclear accumulation of SMAD2 and SMAD3 was significantly higher in *Sirt2*^*tKO*^ UUO kidneys (Fig. 9f, column 4 vs 3). In addition, renal tubule knockout of *Sirt2* up-regulated the expression of TGF-β regulated genes (Fig. 9g). Consistently, AAV-mediated renal tubule SIRT2 overexpression inhibited the phosphorylation and acetylation of SMAD2 and SMAD3, but not the protein level of TGF-β1 (Fig. S8a–c, column 4 vs 3), and reduced the nuclear accumulation of SMAD2 and SMAD3 as well as TGF-β regulated genes (Fig. S8d–f, column 4 vs 3) in the kidneys of UUO mice. In agreement with this, similar results were also observed in uIRI mice (Fig. S5g–j). Taken together, these results indicate that SIRT2 inhibits the activation of SMAD2 and SMAD3.

### Deficiency of SMAD2 and SMAD3 repressed the renal injury mediated by renal tubular epithelial cell-specific deletion of Sirt2 in UUO mice

**Fig. 10:**
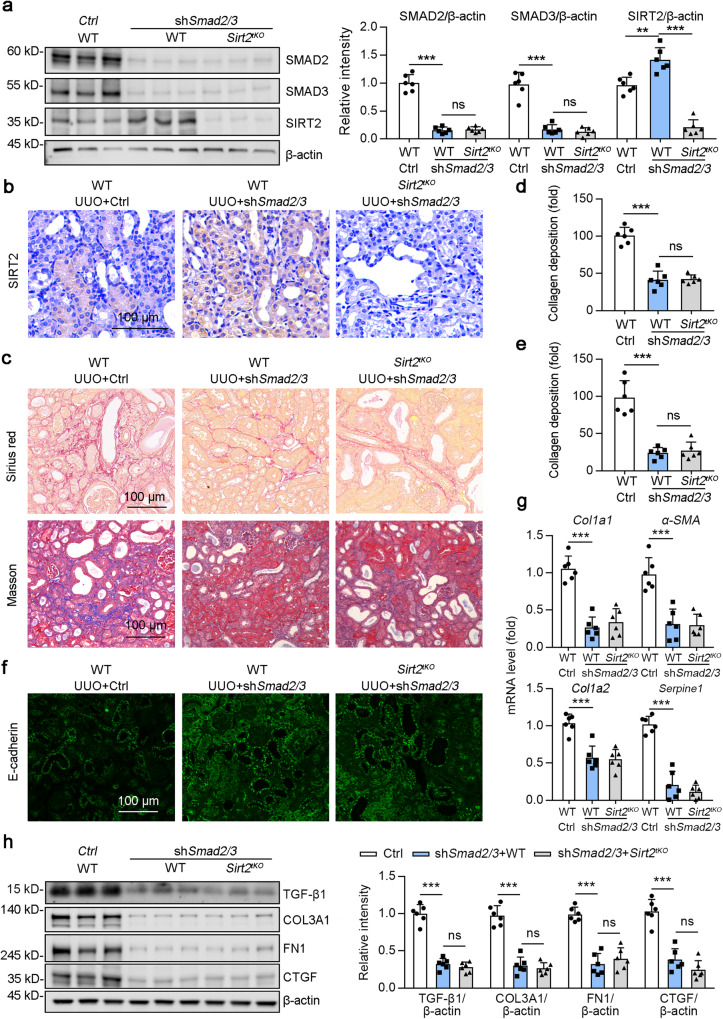
SMAD2/3-deficiency abrogates SIRT2 knockout mediated kidney injury. AAV-Ctrl or AAV-*Ggt* (gamma-glutamyltransferase 1)-*shSmad2/3* was injected into bilateral kidneys of mice in situ at three independent points. After 2-week transfection, mice received UUO surgery, and the contralateral kidneys were used as control. **a** Western blot analysis of SMAD2, SMAD3, SIRT2 and β-actin in the kidneys from mice at day 10 post-surgery, with quantitative results shown in the right panel, and β-actin used as the loading control (*n* = 6). **b** Representative images of SIRT2 immunohistochemical staining in the kidney sections. **c–e** Representative images of Sirius red and Masson’s trichrome staining were shown (**c**), and the collagen deposition (**c** for Sirius red staining and (**d**) for Masson’s trichrome staining) was quantified in the kidney sections in 3 fields per mice at ×100 magnification (*n* = 6). **f** Representative images of E-cadherin immunofluorescence staining were shown in the kidney from mice at day 10 post-surgery. **g** The mRNA level of *Col1a1*, *α-SMA, Col1a2*, and *Serpine1* (*n* = 6). **h** Western blot analysis of COL3A1, FN1, CTGF, and β-actin in the kidneys from mice at day 10 post-surgery, with quantitative results shown in the right panel, and β-actin used as the loading control (*n* = 6). For all panels, data are presented as mean ± SD. *P < 0.05, **P < 0.01, ***P < 0.001 by one-way ANOVA with a Bonferroni correction test.

We further determined whether TECs knockout of SIRT2 in the kidney aggravated obstructive nephropathy in the context of SMAD2 and SMAD3 renal ablation in mice (Fig. 10a, b). AAV9-*Ggt*-*shSmad2/3*-mediated renal tubular epithelial cell-specific *Smad2* and *Smad3* knockdown in UUO mice reduced the collagen deposition (Fig. 10c–e), retarded E-cadherin loss (Fig. 10f), downregulated the mRNA level of *Col1a1* and *α-SMA* (Fig. 10g), and decreased the protein level of COL3A1, FN1 and CTGF (Fig. 10h). More importantly, there were no obvious difference in these changes (collagen deposition and profibrogenic genes expression) between WT and *Sirt2*^*tKO*^ mice transfected with *shSmad2/3*. Overall, these data suggested that SMAD2 and SMAD3 are important targets of SIRT2 against renal fibrosis.

### Persistent TGF-β treatment inhibited transcription levels of SIRT2

Reanalysis of microarray data obtained from the GEO database (GSE70544) showed that there was no significant change in the transcription level of *SIRT2* after hypoxia or cytokine (cytokine cocktail consisting of 50 ng/ml IL-6, 50 ng/ml TNF-α, and 20 ng/ml interferon-γ) stimulation in human PTECs (HKC-8 cells). We found that TGF-β treatment significantly decreased the transcription level of *SIRT2* after 54 h of treatment (Fig. S9a). Epigenetic mechanisms (such as DNA methylation or histone modifications) are known to play a vital role in the regulation of TGF-β in renal fibrosis [43]. Therefore, HK2 cells were incubated with TGF-β and 5-aza (DNA methyltransferase inhibitor) or TSA (specific histone deacetylase type I and II inhibitors), as indicated in the figure. The results showed that only TSA treatment blocked the TGF-β-mediated downregulation of *SIRT2* mRNA levels (Fig. S9b). A time-course experiment further showed prolonged treatment with TGF-β (72–96 h) significantly downregulated SIRT2 expression (Fig. S9c), and this change was blocked by co-treatment with TGF-β and MC1568 (selective inhibitors of class II), but not TGF-β and Pimelic Diphenylamide 106 (class I HDACs; data not shown). Moreover, siRNA-mediated knockdown of HDAC6, HDAC9, or HDAC10, but not HDAC4, HDAC5, and HDAC7, blocked the downregulation of SIRT2 mediated by long-term TGF-β stimulation (Fig. S9d). Taken together, these results suggest that TGF-β may suppress *SIRT2* expression in HK2 cells through epigenetic modifications.

### Specific deletion of Sirt2 in hepatocyte aggravated CCl_4_-induced hepatic fibrosis

Finally, we generated hepatocyte-specific *Sirt2* knockout mice (*Sirt2*^*hKO*^) by crossing *Sirt2*^*fl/fl*^ mice to mice bearing an Albumin-Cre transgene (Fig S10a). Notably, in a model of liver fibrosis induced by CCl4 treatment, specific deletion of *Sirt2* in hepatocyte also displayed a more evident accumulation of collagen protein, high phosphorylation of SMAD2 and SMAD3, and higher expression of fibrotic genes, such as *Serpine1*, *Col1a1*, *Col1a2*, and *Smad7*, compared to that in the control liver (Fig. S10, a–d, *column 4 vs 3*), suggesting that SIRT2 in hepatocytes plays an important role during fibrogenesis not only in the kidney but also in liver tissue.

## Discussion

In the present study, we have demonstrated that SIRT2, an NAD^+^-dependent deacetylase, is increased in the renal tubules and is critical to the pathogenic pathway of renal fibrosis. SIRT2 is downregulated in fibrotic kidneys from patients with DKD and CKD as well as in UUO- or uIRI-induced mouse fibrosis models. Our investigations have revealed that SIRT2 suppresses renal fibrosis by inhibiting pro-fibrotic TGF-β signaling through the promotion of deacetylation of SMAD2 and SMAD3. In addition, chronic activation of TGF-β reduced SIRT2 transcription by histone de-acetylase- (HDAC-) mediated epigenetic silencing in renal tubule epithelial cells. Consistent with the anti-fibrotic effect of SIRT2 observed in vitro, targeted deletion of SIRT2 in renal tubule epithelial cells aggravated renal fibrosis induced by UUO. Thus, specific overexpression of SIRT2 in renal tubule epithelial cells may alleviate and restrain UUO- and uIRI-caused renal fibrosis. Above all, these findings uncover the causal role of SIRT2 in the progression of renal fibrosis through the control of TGF-β/SMAD signaling and suggest that SIRT2 has the potential to be an effective therapeutic approach for both prevention and treatment of renal fibrosis as part of CKD.

SIRT2 functions inconsistently in regulating fibrotic phenotypes in different organs and cells. For instance, global *Sirt2* knockout had a strong exaggerating effect on fibrosis of cardiac tissue in angiotensin II-induced hypertrophic hearts in mice [44]. Mechanistic investigations have demonstrated that SIRT2 binds to and deacetylates LKB1 at lysine 48, enhancing LKB1-AMPK signaling [44]. Consistently, global *Sirt2* knockout has been demonstrated to lead to prolonged fibrogenic responses in cerulein-induced mouse models of acute pancreatitis [45]. Interestingly, the fibrotic phenotype of liver tissue was more obvious in the HFD-fed global *Sirt2* knockout mice compared to WT mice [46]. In addition, NAD^+^-boosting therapy protected against HFD-induced inflammation and fibrosis by activating SIRT2 in hepatocyte [47]. Intriguingly, blocking the activity of sirtuin 2 (SIRT2) using inhibitors or shRNAs significantly suppressed fibrogenic gene expression in hepatic stellate cells [48], indicating a contradictory effect of the fibrogenic gene regulated by SIRT2 even within the same organ. In the present study, a renal tubule epithelial cell-specific *Sirt2* conditional knockout mouse line (*Sirt2*^*tKO*^) was utilized, showing that the induction of SIRT2 deletion in kidney renal tubule epithelial cells 7 days after UUO, a time point when renal fibrosis was well established (Fig. 2a), significantly promoted the progression and expansion of renal fibrosis compared to control mice. Consistently, renal tubule epithelial cell-specific *Sirt2* overexpression alleviated renal fibrosis in UUO and uIRI mice (Figs. 3e and S4a). To the best of our knowledge, this study is the first to suggest an anti-fibrotic role for SIRT2 in renal tubular epithelial cells.

The role of SMAD2 in TGF-β signaling is controversial in the setting of renal fibrosis as SMAD2 renal ablation aggravates fibrosis contrary to expectations [49]. However, another study showed that SIRT6 alleviated liver fibrosis by deacetylating and inactivating SMAD2 in hepatic stellate cells of an experimental liver fibrosis model [17]. Furthermore, curcumin was found to inhibit liver fibrosis in rats by a mechanism that may be related to enhanced ubiquitination and proteasomal degradation of SMAD2 by SMURF2 [34]. In contrast to SMAD2, SMAD3 depletion protects kidneys from fibrosis establishing SMAD3 as a major downstream target of fibrotic TGF-beta signaling [50]. In the current study, SIRT2 overexpression suppressed the acetylation of SMAD2 and SMAD3, collagen deposition, and the expression of fibrogenic genes in the kidneys of UUO mice (Figs. 3 and 9). In addition, we also showed that SIRT2 promoted the ubiquitination of SMAD2 and reduced the protein stability of SMAD2 upon TGF-β stimulation in HK2 cells (Fig. 5c, d), all of which were dependent on SMURF2 (Fig. 7a–g). A previous report showed that it is only in the presence of TGF-β that SMURF2 will interact with endogenous SMAD2 [31]. In agreement with this, we found that SIRT2 overexpression enhanced the binding of SMAD2 and SMURF2 only in the presence of TGF-β (Fig. 7c, d). In addition, overexpression or deletion of *Sirt2* in PTEC did not significantly change the protein level of SMAD2 without TGF-β stimulation (Fig. 4a, d). As for SMAD3, we found that SIRT2 directly interacted with and deacetylated SMAD3 on lys341 and lys378, resulting in reducing the phosphorylation and nuclear localization of SMAD3 (Fig. 8). Importantly, deficiency of SMAD2 and SMAD3 improved the renal fibrosis in UUO mice, and there is no obviously difference in renal fibrosis between WT and *Sirt2*^*tKO*^ mice when SMAD2 and SMAD3 is deficient (Fig. 10). In addition, SMAD2 and SMAD3 knockout augmented the production of inflammatory factor (TNFα and IL-6) [51], while SIRT2-mediated NLRP3 deacetylation protects against inflammation [22], indicating SIRT2 may be a more attractive target to improving renal fibrosis. Hence, we suggest that targeting SIRT2 could restrain the activation of the TGF-β signaling pathway by inhibiting both SMAD2 and SMAD3 activity.

Notably, a recent study found that SIRT2 promotes SMAD2/3 activation in TGF-β-treated lung fibroblasts, contrary to our observation [52]. In addition, according to this research, they found that *Sirt2* expression was upregulated in transforming growth factor-β1 (TGF-β1) treated human embryonic lung fibroblasts [52]. However, to assess the expression of SIRT2 in another fibrotic disease, we reanalyzed the database obtained from liver biopsy specimens from patients with cirrhosis (GSE139602) and lung biopsy specimens from patients with idiopathic pulmonary fibrosis (GSE110147). Intriguingly, we found that SIRT2 transcription levels were also lower in fibrotic liver (logFC = −0.55, *p* = 0.00744) and lung (logFC = −0.32; *p* = 0.0003), respectively, compared to those in HCs (data not shown). Importantly, TGF-β-SMAD2/3 signaling pathway, in addition to its role as a profibrotic factor, is a prohypertrophic factor in the heart [53]. However, Sirt2-KO markedly exaggerated cardiac hypertrophy and fibrosis as well as decreases in cardiac ejection fraction and fractional shortening in aged (24-month-old) mice and Ang II-infused mice, which suggests a contradiction with the fact that SIRT2 helps SMAD2/3 activation [44]. In addition, treatment with AGK2, a selective inhibitor of SIRT2, blocked the therapeutic action of nicotinamide riboside (NR) against NAFLD pathologies and the downregulation of inflammatory and fibrotic factors mediated by NR [47]. If the absence of SIRT2 has a protective effect on fibrosis, then AGK2 and NR should play a superimposed protective effect, rather than blocking the protective effect of NR. In the current study, we found that SIRT2 expression were reduced in the liver of CCL_4_-induced hepatic fibrosis (Fig. S10a), and specific deletion of *Sirt2* in hepatocyte aggravated CCl_4_-induced hepatic fibrosis and upregulated TGF-β regulated genes expression (Fig. S10b–d).

Above all, we have shown that SIRT2 acts as a key checkpoint in controling TGF-β signaling and TEC activation during the development of fibrotic kidney disease. However, it is important to note that chronic activation of TGF-β signaling creates the ability to escape the regulatory effects of SIRT2 by the inhibition of SIRT2 transcription. Overexpression of SIRT2 in TECs can restrain the signaling of TGF-β/SMAD and cease the tissue’s excessive response, thus inhibiting renal fibrosis.

## Methods

### Studies in animals

All Animal care and experimental protocols for in vivo studies conformed to the Guide for the Care and Use of Laboratory Animals published by the National Institutes of Health (NIH; NIH publication no.:85–23, revised 1996) and were approved by the Ethics Committee of the Second Clinical Medical College of Jinan University, Shenzhen People’s Hospital (Shenzhen, China). The sample size for the animal studies was calculated based on a survey of data from published research or preliminary studies. C57BL/6 J mice, Sirt2^−/−^ (C57BL/6N-Sirt2em1cyagen, strain No. KOCMP-64383-Sirt2-B6N-VA), *Sirt2*^*flox/flox*^ (C57BL/6J-Sirt2^em1cyagen^; strain No. CKOCMP-64383-Sirt2-B6J-VA), Albumin-Cre mice (B6/JGpt-H11^em1Cin(Alb-iCre)^/Gpt, strain No. T003814) and *Pax8*-Cre mice (strain No. C001040) based on a C57BL/6J background obtained from Cyagen Biosciences (Guangzhou) Inc (Guangzhou, Guangdong, China). These mice were maintained in SPF units of the Animal Center of Shenzhen People’s Hospital with a 12 h light cycle from 8 a.m. to 8 p.m., 23 ± 1 °C, 60–70% humidity. Mice were allowed to acclimatize to their housing environment for 7 days before the experiments. At the end of the experiments, all mice were anesthetized and euthanized in a CO_2_ chamber, followed by the collection of kidney tissues. All animals were randomized before treatment. Mice were treated in a blinded fashion as the drugs used for treating animals were prepared by researchers who did not carry out the treatments. No mice were excluded from the statistical analysis. Studies were performed in accordance with the German Animal Welfare Act and reporting follows the ARRIVE guidelines [54].

### Mice kidney and liver fibrotic models

Male C57BL/6 mice that were 8–10 weeks old, *Sirt2*^*flox/flox*^, and cell type-specific conditional knockout mice were treated with either UUO or uIRI procedures for renal fibrosis induction according to established methods. Mice were anesthetized by intraperitoneal injection of pentobarbital (50 mg/kg body weight) prior to surgery. For UUO surgery, the left ureter of the mouse was revealed through a left-sided incision, and the ureter was double-ligated using 6-0 silk. A Sham-operated group did not receive ligation. The left kidney was collected at the time of surgery, followed by the euthanization of the mice. As uIRI surgery was performed, the left renal artery was exposed through a left abdominal incision and subsequently clamped with a vascular clamp for 30 min. Thirty minutes later, the vascular clamp was released, and the left kidney returned from dark red to bright red, suggesting successful modelling. As uIRI surgery was performed, a heating pad to be used to maintain a body temperature of 37 °C. Injured and contralateral kidneys were collected 24 day following the operation. Liver fibrosis models were established using CCl_4_. In the CCl4 (Macklin, Shanghai, China; Cat.No.C805329)(1:4 diluted in corn oil)-induced mouse liver fibrosis model, mice were administered CCl4 by i.p. injection at a dose of 5 μL/g body weight every 3 days for 6 weeks (corn oil i.p. injection was used as the control group).

### Sirt2 overepxression and SMAD2/3 knockdown mice

8-week-old mice received in situ renal injection with AAV9- empty vector (AAV9-*Ctrl*; control group), AAV9-*Ggt*-*Sirt2* (*Sirt2* overexpression group; *Ggt*, proximal tubule specific promoter) or AAV9-*Ggt*-sh*Smad2/3* at five independent points (10–15 μl virus per poin; virus injected dose: 2.5 E + 11 v.g.) in the bilateral kidneys of mice (*n* = 6). Adeno-associated virus type 9 constructs, including GV501 empty vector and *Sirt2* (5.1 E + 13 v.g./ml) or sh*Smad2/3* (5.37 E + 12 v.g./ml) were provided by GeneChem Company (Shanghai, China).

After 2 weeks of AAV infection, mice underwent UUO or uIRI surgery, as previously mentioned.

### Human renal specimens

Assessment of histopathology was evaluated in a blinded manner by two experienced pathologists and classified according to the Renal Pathology Society’s Pathologic Classification of Diabetic Kidney Disease on the basis of glomerular pathology. Patients diagnosed with minimal disease change were included in the control group (*n* = 1; eGFR = 142.53). The clinical characteristics of DKD in the biopsy samples are shown in Table S1. Venous blood samples were collected from eight individuals with type 2 diabetes after overnight ( ≥ 10 h) fasting. Fasting blood glucose (FBG) concentrations were measured using an automatic biochemical analyzer (AU5800; Beckman Coulter, USA). Reference ranges were obtained from the central laboratory of Shenzhen People’s Hospital (Shenzhen, China), and the variables measured were within the reference ranges based on age, sex, and ethnicity. This study was approved by the Institutional Review Board and Ethics Committee of the Shenzhen People’s Hospital (approval no. LL-KT-2018338). Written informed consent was obtained from all participants prior to their inclusion in the study.

### Histology

Mouse kidneys were fixed in 4% paraformaldehyde for 24 h, dehydrated, embedded in paraffin, and sectioned into 4 μm thickness for Masson’s trichrome (Cat. No. 1004850001, Sigma-Aldrich, St. Louis, Missouri, USA), picrosirius red (Cat. No. ab245887, Sigma-Aldrich), Periodic AcidSchiff (Cat. No. 101646, Sigma-Aldrich, St. Louis, Missouri, USA), or H&E (Cat. No. ab245880, Abcam, Cambridge, UK.), respectively. The fibrotic area was quantified using ImageJ software (NIH, http://rsbweb.nih.gov/ij/).

### Immunohistochemical (IHC) staining

Paraffin-embedded mouse kidney samples were sliced into 4 μm-thick sections and subjected to IHC staining using a Rabbit two-step detection kit (Rabbit enhanced polymer detection system, ZSGB Bio, Beijing, China). Briefly, mouse kidney sections were deparaffinized, followed by antigen retrieval, treatment with peroxidase block, and incubation with rabbit anti-SIRT2 (1:100, Abcam, Cat. No. ab211033), α-SMA (1:200, Abcam, Cat. No. ab124964), SMAD2 (1:100, Santa Cruz Biotechnology, Cat. No. sc-393312), SMAD3 (1:100, Abcam, Cat. No. ab52903) primary antibody overnight at 4 °C. Tissue sections were then washed and incubated with the reaction-enhancing solution at room temperature for 20 min. After washing three times with phosphate buffer saline (PBS), tissue sections were treated with enhanced enzyme-labeled goat anti-rabbit IgG polymerase for 20 min and then developed with 3,3′ Diaminobenzidine solution, counterstained with hematoxylin, and mounted with mounting medium. The kidney frozen slices from patients with minimal change disease and DKD were fixed with 4% paraformaldehyde for 20 min, incubated with anti-SIRT2 primary antibody overnight at 4 °C, and subjected to immunohistochemistry staining as described above. The stained area was measured using ImageJ software.

### Immunofluorescence (IF) staining

Paraffin-embedded mouse kidney samples were sliced into 4 μm-thick sections and subjected to IF staining according to an established protocol. Briefly, kidney sections were deparaffinized, followed by antigen retrieval, treatment with peroxidase block, and incubation with mouse anti-E-cadherin (1:200, Cat. No. ab231303, Abcam) and primary antibodies overnight at 4 °C. After washing, sections were incubated with Alexa Fluor 488-labeled anti-mouse secondary antibodies in the dark at room temperature for 1 h. After treatment with an anti-fluorescence quencher containing 4′,6-diamidino-2-phenylindole, the sections were washed and mounted with ProLong Gold (Thermo Fisher Scientific, Shanghai, China). The fluorescence staining area was measured using ImageJ software. After treatment, HK2 cells were fixed as necessary in 4% paraformaldehyde for 20 min, permeabilized, and blocked with 0.1% Triton 100 in 2% BSA for 1 h. HK2 cells were then incubated with anti-SMAD2 (1:200, Abcam, Cat. No. 33875) and subjected to IF staining as previously described.

### Cell culture, transfection

Human proximal tubular epithelial cells (HK2 cells) were purchased from American Type Culture Collection (ATCC, Manassas, VA, USA) and maintained in HyClone Minimum Essential Medium (SH30024, Cytiva, Marlborough, Massachusetts, USA) with 10% FBS and 1% penicillin/streptomycin solution in a humidified incubator supplemented with 95% air/5% CO2 at 37 °C. Human embryonic kidney 293 cells (HEK293) were purchased from ATCC and maintained in HyClone Dulbecco’s Modified Eagle Medium (SH30022, Cytiva) with 10% FBS, 1% glutamine, and 1% penicillin/streptomycin solution in a humidified incubator supplemented with 95% air/5 % CO2 at 37 °C. Cells were regularly checked for mycoplasma in a standardized manner, by a qPCR test, performed under ISO17025 accreditation to ensure work was conducted in mycoplasma-negative cells.

Primary mouse renal tubular epithelial cells were isolated using an established protocol. Briefly, the cortex of the kidneys was carefully dissected and chopped into small pieces. Then, 1 mg/ml of collagenase solution was applied and incubated at 37 °C for 30 min with gentle agitation. The digestion was terminated by FBS and then filtered sequentially. Fragmented tubules were collected and maintained in a renal epithelial cell basal medium using a growth kit. The medium was changed for the first time, after 72 h. The purity of the primary mouse renal tubular epithelial cells was confirmed by immunofluorescence staining of SGLT1 (Novus, NBP2-20338). Cells at passages 2-5 were used for the experiments.

Adenovirus particles containing human SIRT2, WT SMAD2, mutant SMAD2 (lysine 451 mutated to arginine; K451R), and P/CAF were provided by GeneChem (Shanghai, China). Adenovirus particles containing catalytic mutants Sirt2-N168A (asparagine 168 mutated to alanine; N168A) and Sirt2-H187Y (histidine 187 mutated to tyrosine; H187Y) were provided by GeneChem Company (Shanghai, China). Adenovirus was used to transfect HK2 cells for 24 h, and adenovirus particles containing empty vectors were used as controls. The dCas9-SMA (Synergistic activation mediator) system was used to constructed lentivirus containing human CBP (GeneChem Company, Shanghai, China). HK2 cells were transfected with lentivirus for 48 h, where lentivirus particles containing empty vectors were used as controls. The commercial siRNA Ctrl (Cat. No. sc-37007, Santa Cruz), siRNA *SIRT2* (sc-40988, Santa Cruz), siRNA *SMURF2* (Cat. No. sc-41675, Santa Cruz), and siRNA *P/CAF* (Cat. No. sc-36198, Santa Cruz) were transfected using Lipofectamine 3000 (Invitrogen, Carlsbad, California, USA) according to the manufacturer’s instructions.

### Quantitative real-time PCR (qPCR)

Total RNA was extracted from cells and kidney tissue using TRIzol reagent (Invitrogen), according to the manufacturer’s protocols. PrimeScript™ RT reagent Kit with gDNA Eraser (TaKaRa, Kusatsu, Shiga, Japan) was used for RNA reverse transcription, and SYBR Green–based qRT-PCR was performed using the ABI StepOnePlus™ Real-time PCR system (Applied Biosystems, Waltham, Massachussetts, USA). The PCR primers used are listed in Supplementary Table S2. We analyzed the relative mRNA levels of the target genes using the equation 2^−ΔΔCT^, with β-actin used as a housekeeping gene. Normalization using the level detected in the control samples was 1.

### Western blotting

After treatment, the cells and mouse kidney tissues were homogenized in RIPA lysis buffer (Cat. No. R0010, Solarbio, Beijing, China) supplemented with protease inhibitors (Cat. No. P6730, Solarbio) and PMSF (Cat. No. R0010, Solarbio). After being centrifuged for 5 min (10,000 *g*) at 4 °C, the supernatant was collected, and the protein concentrations were quantified using Pierce BCA Protein Assay Kit (Cat. No. 23225, Thermo Fisher Scientific). Protein lysates were mixed with 5× protein loading buffer and boiled for 5 min at 95 °C. Protein samples were fractionated on 4–12% SDS-PAGE gel, transferred to PVDF membranes, and blocked in blocking buffer (5% fresh dry fat-free milk, 0.1% Tween 20 in TBS). Membranes were incubated with primary antibodies (list provided below) overnight at 4 °C. Blots were developed using HRP-conjugated anti-mouse or anti-rabbit IgG secondary antibodies (1:10000, Abcam) and WesternBright ECL HRP substrate (Advansta San Jose, CA, USA). Densitometry analysis was performed using Quantity One Software (Bio-Rad Laboratories, Hercules, CA), and the protein levels were normalized to β-actin. The details of full length western blots were shown in ‘Supplemental Material’ file.

### Antibodies used in Western blottings

Antibodies used included: SIRT2 (Abcam, Cat. No. ab211033), CTGF (Abcam, Cat. No. ab6992), FN1 (ABclonal, Wuhan, China; Cat. No. A12977), COL1A1 (Santa Cruz, Cat. No. sc-59772), TGF-β1 (Abcam, Cat. No. ab92486), β-actin (bioss, Beijing, China; Cat. No. bs-0061R), COL3A1 (Santa Cruz, Cat. No. sc-514601), E-cadherin (BD Biosciences, Cat. No. 610182), α-SMA (Santa Cruz, Cat. No. sc-84326), p-SMAD2 (Cell Signaling Technology, Cat. No. 18338), SMAD2 (Abcam, Cat. No. ab40855), acetyl Lysine (Abcam, Cat. No. ab190479), Ubiquitin (Novus, Cat. No. NB300-129), SMURF2 (ABclonal, Cat. No. A2278), CREBBP (ABclonal, Cat. No. A14237), P/CAF (ABclonal, Cat. No. A0066), Goat Anti-Rabbit IgG H&L (HRP) (Abcam, Cat. No. ab6721), and goat anti-mouse IgG H&L (HRP) (Abcam, Cat. No. ab205719).

### Coimmunoprecipitation (Co-IP)

After treatment, cells were lysed in an ice-cold co-IP buffer (20 mM Tris-HCl, pH 8.0, 100 mM NaCl, 1 mM EDTA, and 0.5% NP-40) containing a protease inhibitor cocktail (04693132001, Roche, Basel, Switzerland) for 30 min. The cell homogenates were then centrifuged at 13,000 × *g* for 15 min. The supernatant was incubated with the primary antibody (anti-SMAD2 [Cat. No. ab40855, Abcam), anti-SIRT2 (Cat. No. ab211033, Abcam), anti-IgG] on a rocking at 4 °C overnight. To ensure saturation of the primary antibody, we cultured and collected adequate cell lysates for IP. The antibody-protein mixture was incubated with protein A/G-agarose beads (Cat. No. 78610) at 4 °C for 3 h. The beads were washed five to six times with cold IP buffer and resuspended in loading buffer. The cell lysates and immunoprecipitates were denatured in loading buffer at 95 °C for 5 min, and western blotting analysis was performed.

### Luciferase reporter assay for TGF-β activity

For assays involving TGF-β stimulation, using the effectene transfection protocol, the cells were transfected with a p3TP-Luc reporter plasmid containing PAI-1 promoter elements. Transfection efficiency was determined by co-transfection with the β-gal reporter plasmid. The transfection mixture was removed 24 h later and the medium was replaced with fresh medium with or without TGF-β (2 ng/ml). The luciferase and galactosidase activities were measured after 24 h. All assays were performed in triplicate.

### Isolation of Cytoplasmic or nuclear protein

Cytoplasmic or nuclear extracts were prepared from HK2 cells or kidneys using a cytoplasmic and nuclear protein isolation kit (Cat. No 78833, Thermo Fisher Scientific). Briefly, the tube was vortexed vigorously in the highest setting for 15 s to fully suspend the cell pellet (or tissues were homogenized in PBS). The tubes were incubated on ice for 10 min. Next, ice-cold cytoplasmic extraction reagent II solution was added to the tubes. The tube was vortexed and incubated on ice. The tube was centrifuged for 5 min at maximum speed in a microcentrifuge ( ~ 16,000 × *g*), and the supernatant (cytoplasmic extract) was transferred to a tube. The insoluble (pellet) fraction, which contains nuclei, was suspended in ice-cold nuclear extraction reagent and then vortexed at the highest setting for 15 s. The sample was placed on ice and vortexed for 15 s every 10 min for 40 min. The tube was centrifuged at maximum speed ( ~ 16,000 × g) in a microcentrifuge for 10 min, and the supernatant (nuclear extract) fraction was transferred to a tube.

### Protein stability assay

SMAD2 protein stability was measured by a protein stability assay. Ad-null, Ad-*SIRT2*, siCtrl or si*P/CAF* was transfected with HK2 cells for 24 h, and then treated with 100 μg/mL CHX to block protein translation. Immunoblotting at 0, 4, and 8 h was used to quantify SMAD2 protein levels after CHX treatment. These were expressed as percentages relative to time 0. In addition, HK2 cells transfected with Ad-Null, Ad-*SIRT2*, siCtrl, or si*SIRT2* for 24 h were incubated with or without 20 μM MG132 for 8 h under TGF-β stimulation, and SMAD2 protein levels were quantified by immunoblotting.

### Measurements of renal functions

Twenty-four-hour urine samples collected were measured for daily urinary albumin excretion rate (mg/day) using ELISA (Alpha Diagnostics, Burlington, North Carolina, USA). Urinary albumin-to-creatinine ratio was calculated as urine albumin/urine creatinine (μg/mg), and urinary creatinine measured using an ELISA kit for mouse albumin (Exocell, Philadelphia, Pennsylvania, USA).

### Reanalysis of data obtained from GEO and Nephroseq databases

Transcriptome data (GSE30122) from DKD kidney tissue (Glomeruli and Tubuli) in GEO (Gene Expression Omnibus, https://www.ncbi.nlm.nih.gov/geo/) were used for differential gene analysis (log_2_ fold change ≥ 0.5, *P* value < 0.05). Calculations and visualization were performed via the “ggplot2”, “ggrepel” and “ggthemes” packages of R software (these packages can be downloaded from the following website: https://cran.r-project.org/web/packages/available_packages_by_name.html). *P* values were calculated using the Wald test. Nephroseq (https://nephroseq.org/) provides transcriptomic data for chronic kidney disease, with computational features including differential gene analysis and correlation analysis. Gene expression data were downloaded, re-analyzed, and visualized using R software or GraphPad.

### Protein-protein docking simulation

To determine the strength of the binding ability between of these two proteins and SIRT2, we downloaded their protein structures from the Uniprot database (https://www.uniprot.org/), and found that the △G (Kcal/mol) of SIRT2 and SMAD2 and SMAD3 were −16.6 and −7, respectively (Fig. S6b). Visual protein-protein docking of SIRT2 and SMAD2 (Fig. 6a), SMURF2 and SMAD2 (Fig. 7e), CBP and SMAD2 (Fig. 8a), and P/CAF and SMAD2 (Fig. 8c) was performed online using HDOCK (http://hdock.phys.hust.edu.cn/).

### Analysis of the conservation of lysine 451 in SMAD2

A gene panel of the NCBI database (https://www.ncbi.nlm.nih.gov/gene/) was used to download amino acid sequences of proteins from multiple species and subsequently compare sequence conservation between species using the UGENE software (version 39; the software can be downloaded from the following website: http://ugene.net/).

### Bioinformatics methods predict acetylase and deacetylase of Lys451 in SMAD2

A Group-based Prediction System (GPS) online tool (http://pail.biocuckoo.org) was used to predict Lys451 in SMAD2 modified by histone acetyltransferases, with high score values suggesting better results. The amino acid sequence of the protein in FASTA format was downloaded from UniProt and uploaded to the GPS website. Deep-PLA (http://deeppla.cancerbio.info/webserver.php) predicted that Lys451 in SMAD2 is modified by histone deacetylase.

### STRING analysis of SIRT2

We searched for renal fibrosis through Geneshot PubMed and Tagger Literature Gene-Gene Co-Mention Matrix in the ENRICHR database (https://maayanlab.cloud/Enrichr/) and identified the top 20 genes that were mostly co-mentioned with renal fibrosis. Subsequently, SIRT2 and these 20 genes were used to construct a protein-protein interaction network using the STRING database (https://string-db.org/), and the proteins predicted to most likely interact with SIRT2 were SMAD2 and SMAD3 (Fig. S6a).

### Isolation of mouse primary hepatocytes

Mouse primary hepatocytes were isolated using a modified two-step collagenase perfusion technique. Briefly, the liver specimen was cannulated under sterile conditions and flushed once with 50 ml washing buffer containing 2.5 mM EGTA (Sigma-Aldrich). This was followed by perfusion with 30 ml digestion buffer containing 0.03% w/v collagenase (Roche Diagnostics) allowing recirculation of the perfusate. The resulting cell suspension was poured through a 100 μM Cell Strainer and centrifuged with subsequent washing of the cell pellet using Serum-free medium (50 g, 2 min, 4 °C). Cells were then re-suspended in William’s medium E (Biochrom AG) containing 10% fetal bovine serum. Cell number and viability were determined by the Trypan blue exclusion test. Hepatocytes were cultured using six-well plates precoated with a single layer of rat tail collagen. Cells were seeded at a concentration of 2.5 × 10^6^ viable cells per well. Sixteen to eighteen hours later, culture medium were changed to remove dead and nonadherent cells.

### Statistical analysis

Three independent experiments generated the data for this study. SPSS 22.0 software was used to subject raw data to a normal distribution and analysis by 1- sample K-S of a nonparametric test. Values are presented as mean ± SD. Statistical analysis was performed using GraphPad Prism 7.04 (GraphPad Software, San Diego, CA). A two-tailed unpaired Student’s *t* test was performed to compare groups for the animal and cellular experiments with two groups. For more than two groups, a one-way ANOVA followed by a Bonferroni’s post-hoc test was used. A Spearman correlation test was used to calculate correlation coefficients. All statistical analyses were performed blind to avoid bias. Statistical significance was indicated at *P < 0.05, **P < 0.01, and ***P < 0.001.

### Supplementary information

supplementary figure and table

Full and uncropped western blots

aj-checklist

## Data Availability

The original contributions presented in the study are included in the paper/Supplementary Material; further inquiries can be directed to the corresponding author.

## References

[1] Ma Z, Wei Q, Zhang M, Chen J, Dong Z (2018). Dicer deficiency in proximal tubules exacerbates renal injury and tubulointerstitial fibrosis and upregulates Smad2/3. Am. J. Physiol. Renal Physiol.

[2] Liu Y (2011). Cellular and molecular mechanisms of renal fibrosis. Nat. Rev. Nephrol..

[3] Lu YA, Liao CT, Raybould R, Talabani B, Grigorieva I, Szomolay B (2021). Single-Nucleus RNA Sequencing Identifies New Classes of Proximal Tubular Epithelial Cells in Kidney Fibrosis. J. Am. Soc. Nephrol..

[4] Sato Y, Takahashi M, Yanagita M (2020). Pathophysiology of AKI to CKD progression. Semin. Nephrol..

[5] Takaori K, Nakamura J, Yamamoto S, Nakata H, Sato Y, Takase M (2016). Severity and Frequency of Proximal Tubule Injury Determines Renal Prognosis. J. Am. Soc. Nephrol..

[6] Grgic I, Campanholle G, Bijol V, Wang C, Sabbisetti VS, Ichimura T (2012). Targeted proximal tubule injury triggers interstitial fibrosis and glomerulosclerosis. Kidney Int.

[7] Gilbert RE, Cooper ME (1999). The tubulointerstitium in progressive diabetic kidney disease: more than an aftermath of glomerular injury?. Kidney Int.

[8] Anorga S, Overstreet JM, Falke LL, Tang J, Goldschmeding RG, Higgins PJ (2018). Deregulation of Hippo-TAZ pathway during renal injury confers a fibrotic maladaptive phenotype. Faseb J.

[9] Liu R, Zhong Y, Li X, Chen H, Jim B, Zhou MM (2014). Role of transcription factor acetylation in diabetic kidney disease. Diabetes.

[10] Ruggenenti P, Cravedi P, Remuzzi G (2010). The RAAS in the pathogenesis and treatment of diabetic nephropathy. Nat. Rev. Nephrol..

[11] Böttinger EP, Bitzer M (2002). TGF-beta signaling in renal disease. J. Am. Soc. Nephrol..

[12] García-Sánchez O, López-Hernández FJ, López-Novoa JM (2010). An integrative view on the role of TGF-beta in the progressive tubular deletion associated with chronic kidney disease. Kidney Int.

[13] Heldin CH, Miyazono K, ten Dijke P (1997). TGF-beta signalling from cell membrane to nucleus through SMAD proteins. Nature.

[14] Runyan C, Schnaper H, Poncelet A (2004). The phosphatidylinositol 3-kinase/Akt pathway enhances Smad3-stimulated mesangial cell collagen I expression in response to transforming growth factor-beta1. J. Biol. Chem..

[15] Hu B, Wu Z, Phan S (2003). Smad3 mediates transforming growth factor-beta-induced alpha-smooth muscle actin expression. Am. J. Respir. Cell Mol. Biol..

[16] Wrighton K, Feng X (2008). To (TGF)beta or not to (TGF)beta: fine-tuning of Smad signaling via post-translational modifications. Cell. Signalling.

[17] Zhang J, Li Y, Liu Q, Huang Y, Li R, Wu T (2021). Sirt6 Alleviated Liver Fibrosis by Deacetylating Conserved Lysine 54 on Smad2 in Hepatic Stellate Cells. Hepatology (Baltimore, Md).

[18] Simonsson M, Kanduri M, Grönroos E, Heldin C, Ericsson J (2006). The DNA binding activities of Smad2 and Smad3 are regulated by coactivator-mediated acetylation. J. Biol. Chem..

[19] Gomes P, Fleming Outeiro T, Cavadas C (2015). Emerging Role of Sirtuin 2 in the Regulation of Mammalian Metabolism. Trends Pharmacol. Sci..

[20] Michan S, Sinclair D (2007). Sirtuins in mammals: insights into their biological function. Biochem. J.

[21] Lemos V, de Oliveira R, Naia L, Szegö É, Ramos E, Pinho S (2017). The NAD+-dependent deacetylase SIRT2 attenuates oxidative stress and mitochondrial dysfunction and improves insulin sensitivity in hepatocytes. Human Mol. Genet..

[22] He M, Chiang H, Luo H, Zheng Z, Qiao Q, Wang L (2020). An Acetylation Switch of the NLRP3 Inflammasome Regulates Aging-Associated Chronic Inflammation and Insulin Resistance. Cell Metab.

[23] Jung Y, Lee A, Nguyen-Thanh T, Kim D, Kang K, Lee S (2015). SIRT2 Regulates LPS-Induced Renal Tubular CXCL2 and CCL2 Expression. J. Am. Soc. Nephrol.

[24] He W, Dai C, Li Y, Zeng G, Monga S, Liu Y (2009). Wnt/beta-catenin signaling promotes renal interstitial fibrosis. J. Am. Soc. Nephrol..

[25] Takada M, Nadeau K, Shaw G, Tilney N (1997). Prevention of late renal changes after initial ischemia/reperfusion injury by blocking early selectin binding. Transplantation.

[26] Lian Y, Zhou Q, Zhang Y, Zheng F (2011). VEGF ameliorates tubulointerstitial fibrosis in unilateral ureteral obstruction mice via inhibition of epithelial-mesenchymal transition. Acta Pharmacol. Sinica.

[27] Vidyasagar A, Reese S, Hafez O, Huang L, Swain W, Jacobson L (2013). Tubular expression of heat-shock protein 27 inhibits fibrogenesis in obstructive nephropathy. Kidney Int.

[28] Liu F (2008). PCTA: a new player in TGF-beta signaling. Sci. Signaling.

[29] Tovchigrechko A, Vakser I (2006). GRAMM-X public web server for protein-protein docking. Nucleic Acids Res..

[30] Lo R, Massagué J (1999). Ubiquitin-dependent degradation of TGF-beta-activated smad2. Nat. Cell Biol.

[31] Lin X, Liang M, Feng X (2000). Smurf2 is a ubiquitin E3 ligase mediating proteasome-dependent degradation of Smad2 in transforming growth factor-beta signaling. J. Biol. Chem.

[32] Bonni S, Wang H, Causing C, Kavsak P, Stroschein S, Luo K (2001). TGF-beta induces assembly of a Smad2-Smurf2 ubiquitin ligase complex that targets SnoN for degradation. Nat. Cell Biol.

[33] Song Y, Li J, Xie X, Wang H, Li Q (2011). Effects of amlodipine on TGF-β-induced Smad2, 4 expressions in adriamycin toxicity of rat mesangial cells. Arch. Toxicol.

[34] Kong D, Zhang Z, Chen L, Huang W, Zhang F, Wang L (2020). Curcumin blunts epithelial-mesenchymal transition of hepatocytes to alleviate hepatic fibrosis through regulating oxidative stress and autophagy. Redox Biol.

[35] Wrana J, Attisano L, Cárcamo J, Zentella A, Doody J, Laiho M (1992). TGF beta signals through a heteromeric protein kinase receptor complex. Cell.

[36] Caron C, Boyault C, Khochbin S (2005). Regulatory cross-talk between lysine acetylation and ubiquitination: role in the control of protein stability. BioEssays: News Rev. Mol. Cell. Dev. Biol.

[37] Li Y, Dai D, Lu Q, Fei M, Li M, Wu X (2013). Sirt2 suppresses glioma cell growth through targeting NF-κB-miR-21 axis. Biochem. Biophys. Res. Commun.

[38] Suzuki K, Koike T (2007). Resveratrol abolishes resistance to axonal degeneration in slow Wallerian degeneration (WldS) mice: activation of SIRT2, an NAD-dependent tubulin deacetylase. Biochem. Biophys. Res. Commun.

[39] Tu A, Luo K (2007). Acetylation of Smad2 by the co-activator p300 regulates activin and transforming growth factor beta response. J. Biol. Chem.

[40] Chen Y, Zhao W, Yang J, Cheng Z, Luo H, Lu Z (2012). Quantitative acetylome analysis reveals the roles of SIRT1 in regulating diverse substrates and cellular pathways. Mol. Cell. Proteom.

[41] Tan R, He W, Lin X, Kiss L, Liu Y (2008). Smad ubiquitination regulatory factor-2 in the fibrotic kidney: regulation, target specificity, and functional implication. Am. J. Physiol. Renal Physiol.

[42] Imoto S, Sugiyama K, Sekine Y, Matsuda T (2005). Roles for lysine residues of the MH2 domain of Smad3 in transforming growth factor-beta signaling. FEBS Lett.

[43] Meng X, Nikolic-Paterson D, Lan H (2016). TGF-β: the master regulator of fibrosis. Nat. Rev. Nephrol.

[44] Tang X, Chen X, Wang N, Wang X, Liang S, Zheng W (2017). SIRT2 Acts as a Cardioprotective Deacetylase in Pathological Cardiac Hypertrophy. Circulation.

[45] Quan S, Principe D, Dean A, Park S, Grippo P, Gius D (2018). Loss of Sirt2 increases and prolongs a caerulein-induced pancreatitis permissive phenotype and induces spontaneous oncogenic Kras mutations in mice. Sci. Rep.

[46] Park S, Chung M, Son J, Yun H, Park J, Yim J (2021). The role of Sirtuin 2 in sustaining functional integrity of the liver. Life Sci.

[47] Li D, Sun S, Fu J, Ouyang S, Zhao Q, Su L (2021). NAD-boosting therapy alleviates nonalcoholic fatty liver disease via stimulating a novel exerkine Fndc5/irisin. Theranostics.

[48] Arteaga M, Shang N, Ding X, Yong S, Cotler S, Denning M (2016). Inhibition of SIRT2 suppresses hepatic fibrosis. Am. J. Physiol. Gastrointestinal Liver Physiol.

[49] Meng X, Huang X, Chung A, Qin W, Shao X, Igarashi P (2010). Smad2 protects against TGF-beta/Smad3-mediated renal fibrosis. J. Am. Soc. Nephrol.

[50] Sato M, Muragaki Y, Saika S, Roberts A, Ooshima A (2003). Targeted disruption of TGF-beta1/Smad3 signaling protects against renal tubulointerstitial fibrosis induced by unilateral ureteral obstruction. J. Clin. Investig.

[51] Sugiyama Y, Kakoi K, Kimura A, Takada I, Kashiwagi I, Wakabayashi Y (2012). Smad2 and Smad3 are redundantly essential for the suppression of iNOS synthesis in macrophages by regulating IRF3 and STAT1 pathways. Int. Immunol.

[52] Gong H, Zheng C, Lyu X, Dong L, Tan S, Zhang X (2021). viaInhibition of Sirt2 Alleviates Fibroblasts Activation and Pulmonary Fibrosis Smad2/3 Pathway. Front. Pharmacol.

[53] Khalil H, Kanisicak O, Prasad V, Correll R, Fu X, Schips T (2017). Fibroblast-specific TGF-β-Smad2/3 signaling underlies cardiac fibrosis. J. Clin. Investig.

[54] Kilkenny C, Browne W, Cuthill I, Emerson M, Altman D (2010). Improving bioscience research reporting: the ARRIVE guidelines for reporting animal research. PLoS Biol.

