# Supplementary material for: SIRT2 alleviated renal fibrosis by deacetylating SMAD2 and SMAD3 in renal tubular epithelial cells
Source: Cell Death Dis. 2023 Sep 30;14(9):646. doi: 10.1038/s41419-023-06169-1 (PMC10542381; doi:10.1038/s41419-023-06169-1)
Supplement: Supplementary file 1 — supplementary figure and table [file 41419_2023_6169_MOESM1_ESM.docx]

**Electronic Supplementary Figures and Tables**

**Supplementary Figures S1 – S10.**


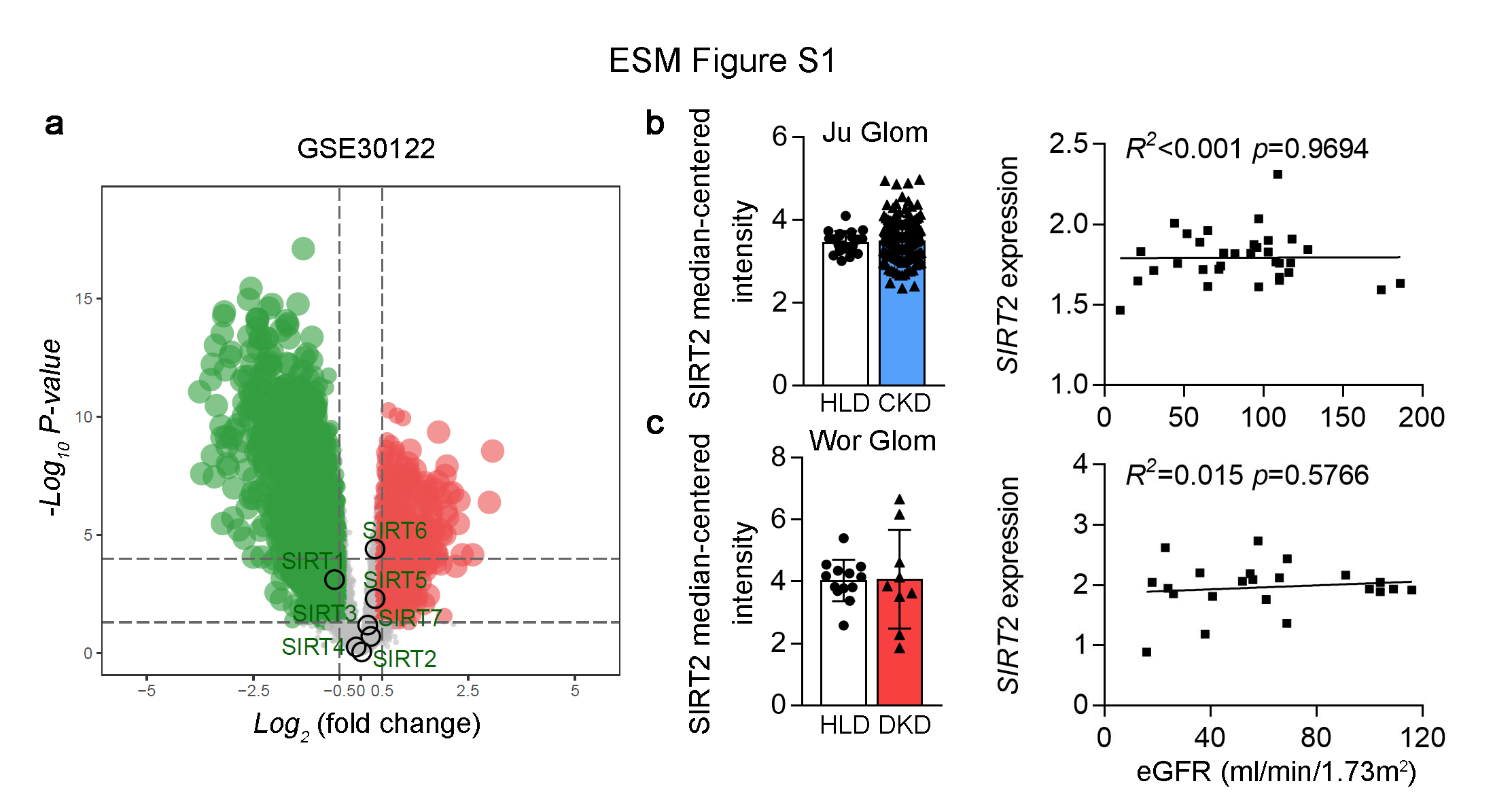


**Figure S1. *SIRT2* was not change in the glomerular of the patients with DKD compared to healthy living donor. (a)** Reanalyzed the database obtained from GEO database (GSE30122) on human kidney specimens from healthy living donor (HLD) and patients with DKD (HLD n = 11, DKD n = 10) showed that *SIRT2* was not significant change in the glomerulus (Glom) from DKD patients compared to those in HLD. **(b,c)** Reanalyzed the database obtained from Nephrin database showed that *SIRT2* (left panel) was not significantly changed in the Glom from HLD and CKD (HLD, n = 21, CKD, n = 178), or HLD and DKD (HLD, n = 13, DKD, n= 9) patients, respectively. There was no linear correlation between SIRT2 expression and eGFR (right panel). For all panels, data are presented as mean ± SD. **P* < 0.05, ***P* < 0.01, ****P* < 0.001 by one-way ANOVA with Bonferroni correction test.


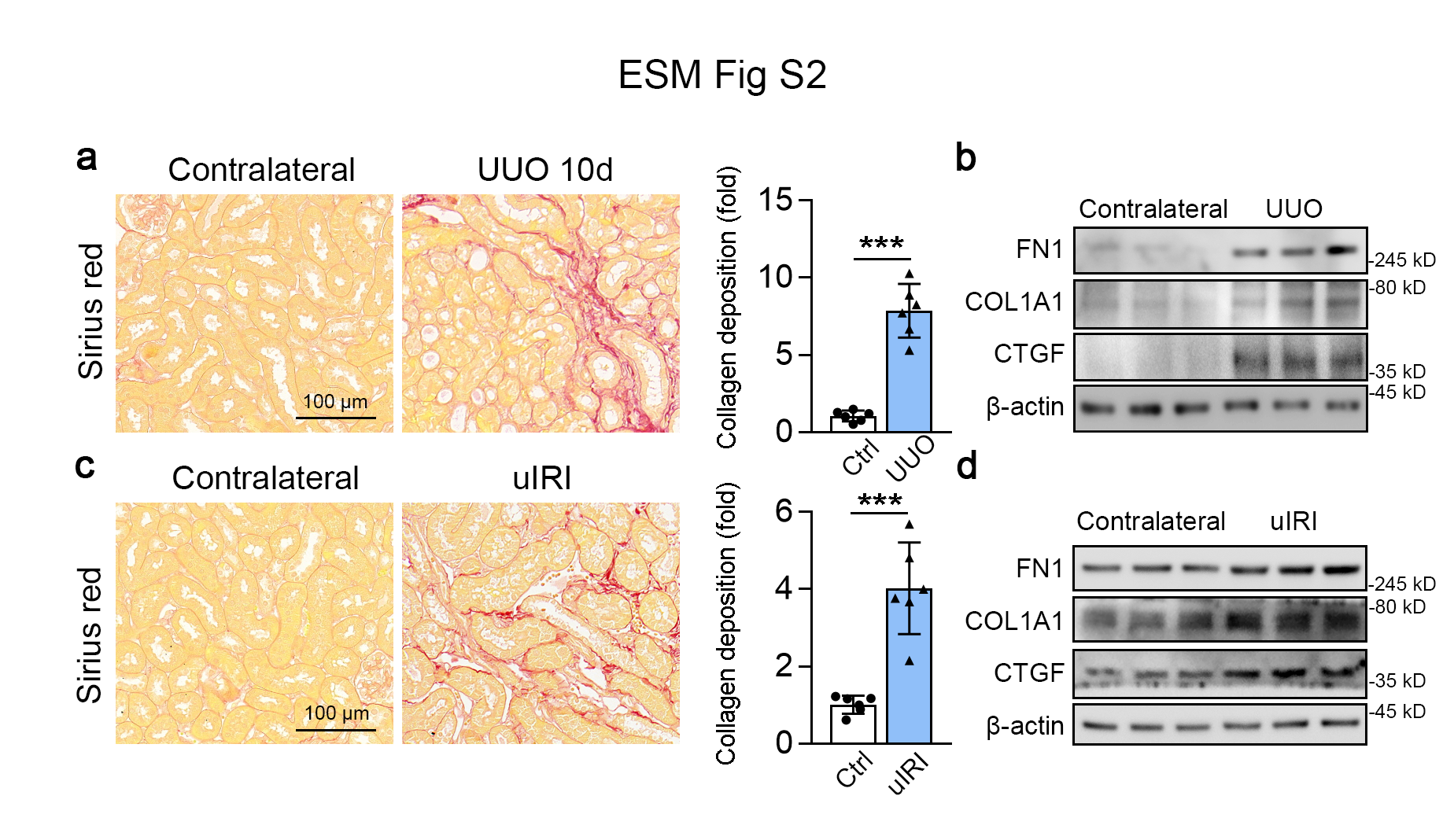


**Figure S2. Evident fibrosis was observed in the kidney of mice after UUO and uIRI surgery. (a to d)** Representative images of Sirius red staining in mouse fibrotic kidneys induced by UUO (a) or uIRI (c) compared with contralateral kidneys were shown (scale bar = 100 μm). The collagen deposition was quantified in the kidney sections in 3 fields per mice at 100× magnification (n = 6). Western blot analysis of FN1, COL1A1, CTGF, and β-actin in fibrotic kidneys induced by UUO (b) or uIRI (d) compared with contralateral kidneys were shown (n = 6). For all panels, data are presented as mean ± SD. **P* < 0.05, ***P* < 0.01, ****P* < 0.001 by one-way ANOVA with Bonferroni correction test.


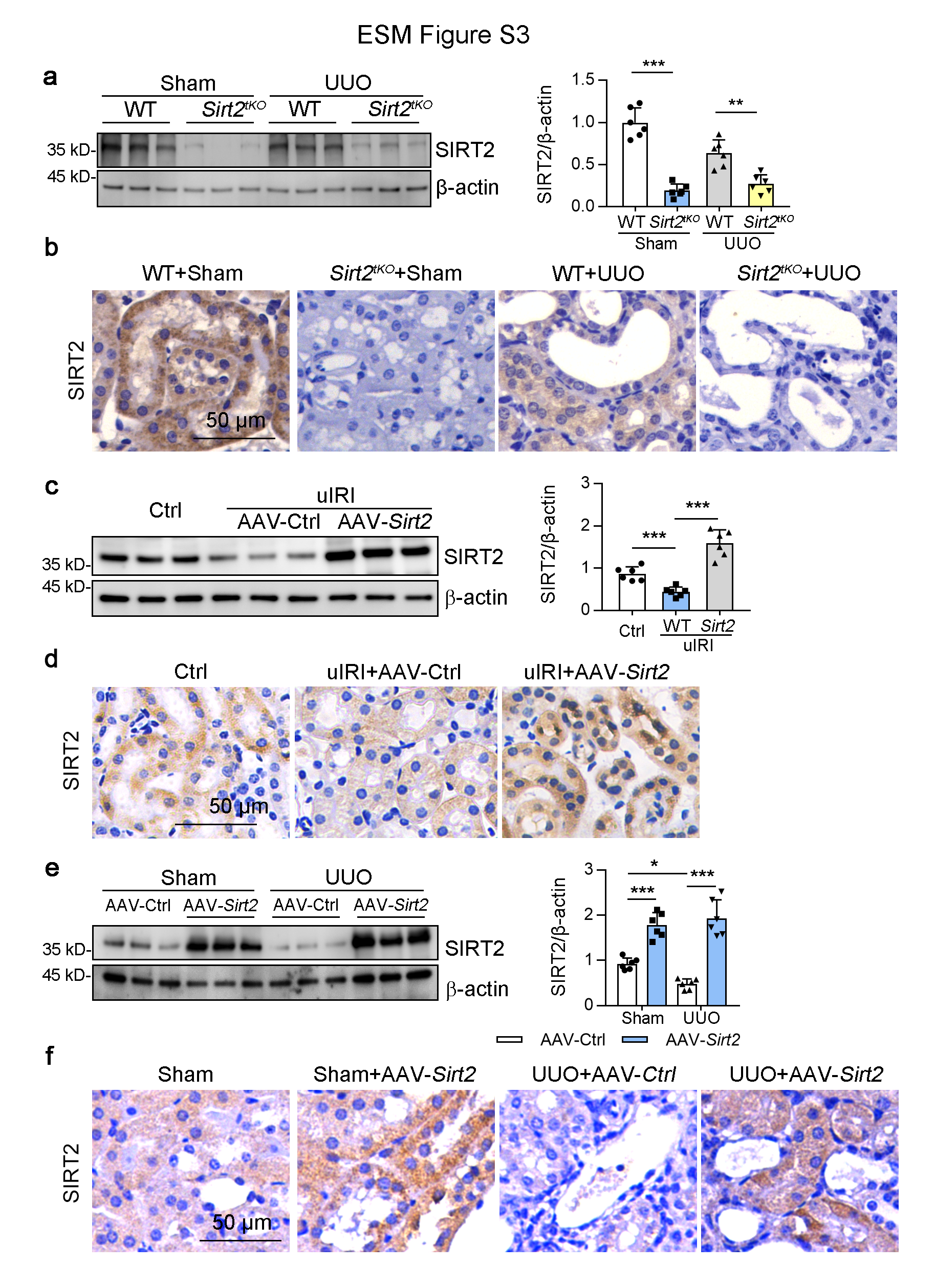


**Figure S3. Detection of SIRT2 expression in kidney in various models. (a,b)** The kidney samples from WT and *Sirt2^tKO^* mice at day 7 post-surgery of UUO. (a) Western blot analysis of SIRT2 and β-actin in kidney, and quantitative results are shown in the right panel, β-actin was used as the loading control (n = 6). (b) Representative images of SIRT2 immunohistochemical staining in the kidney sections. **(c,d)** AAV-Ctrl or AAV-*Ggt* (gamma-glutamyltransferase 1)-*Sirt2* transfected with C57BL/6J mice. After 2-week transfection, mice received uIRI surgery. The kidney samples from mice at 24 d post-surgery of uIRI. (c) Western blot analysis of SIRT2 and β-actin in kidney, and quantitative results were shown in the right panel, β-actin was used as the loading control (n = 6). (d) Representative images of SIRT2 immunohistochemical staining in the kidney sections. **(e, f)** AAV-Ctrl or AAV-*Ggt* (gamma-glutamyltransferase 1)-*Sirt2* was injected into bilateral kidneys of mice in situ at five independent points. After 2-week transfection, mice received UUO surgery, and the contralateral kidneys were used as control. (e) Western blot analysis of SIRT2 and β-actin in kidney, and quantitative results were shown in the right panel, β-actin was used as the loading control (n = 6). (f) Representative images of SIRT2 immunohistochemical staining in the kidney sections. For all panels, data are presented as mean ± SD. **P* < 0.05, ***P* < 0.01, ****P* < 0.001 by one-way ANOVA with Bonferroni correction test.


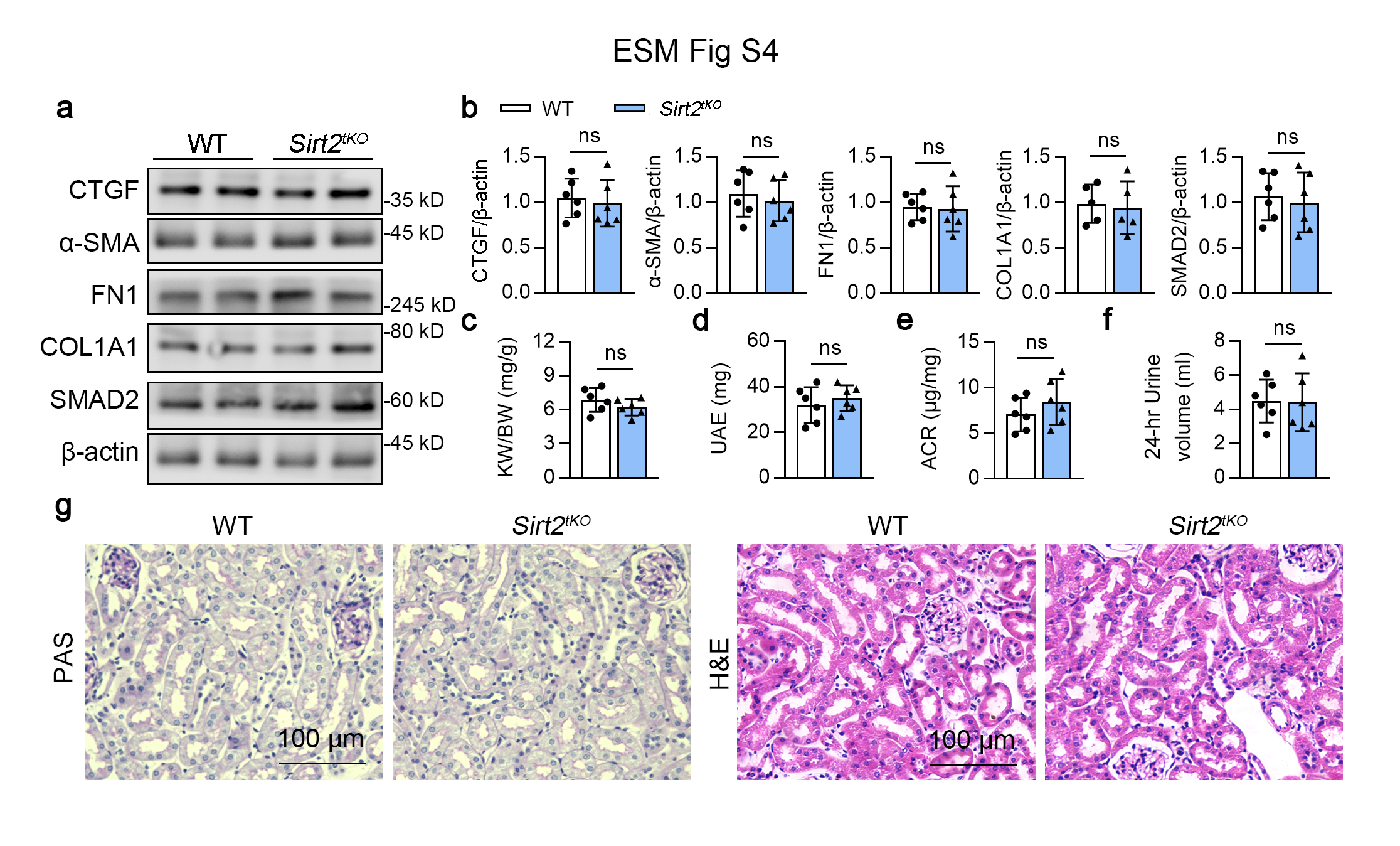


**Figure S4. The renal structure and functions, levels of fibrosis factors in *Sirt2^tKO^* kidneys were not altered under normal conditions.** The kidney samples obtained from 10-week-old WT or *Sirt2^tKO^* mice. **(a)** Western blot analysis of CTGF, α-SMA, FN1, COL1A1, SMAD2, and β-actin. **(b)** Quantitative results of panel a were shown, β-actin was used as the loading control (n = 6). **(c to f)** The ratio of kidney weight (KW, g) and body weight (BW, g), urinary albumin excretion rate (UAE, mg), albumin to creatinine ratio (ACR; μg/mg), and 24-hr Urine volume (ml) (n=6). **(g)** Representative images of PAS and H&E staining in the kidney sections. For all panels, data are presented as mean ± SD. ns (no significance) by one-way ANOVA with Bonferroni correction test.


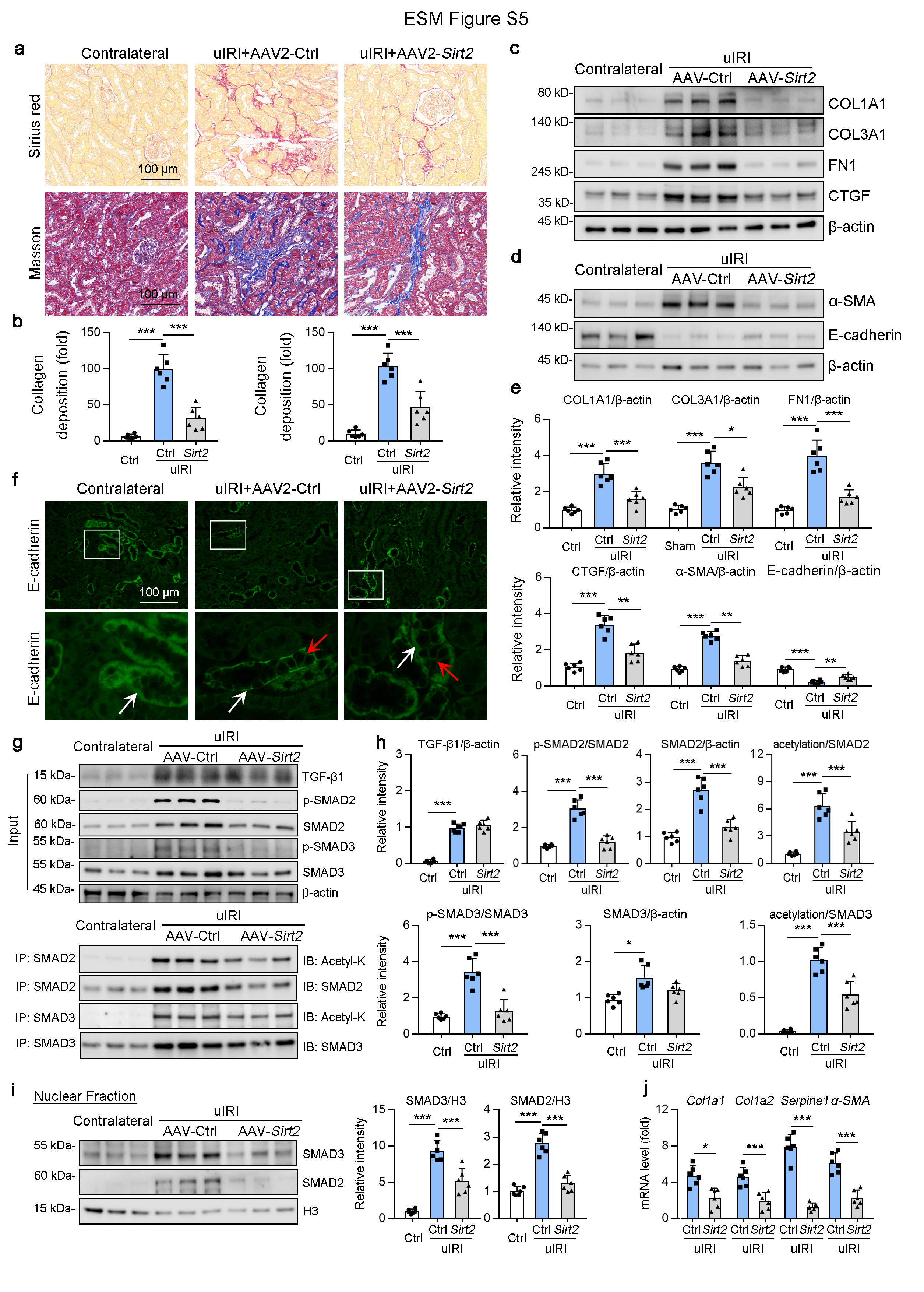


**Figure S5. Renal tubular SIRT2 overexpression alleviated uIRI-induced renal fibrosis.** AAV-Ctrl or AAV-*Ggt* (gamma-glutamyltransferase 1)-*Sirt2* was injected into bilateral kidneys of mice in situ at five independent points. After 2-week transfection, mice received uIRI surgery, and the contralateral kidneys were used as control. **(a,b)** Representative images of Sirius red and Masson’s trichrome staining were shown (a), and the collagen deposition (left for Sirius red staining and right for Masson’s trichrome staining) was quantified in the kidney sections in 3 fields per mice at 100× magnification (n = 6). **(c to e)** (c) Western blot analysis of COL1A1, COL3A1, CTGF, FN1, and β-actin in the kidneys from mice at 24 d post-surgery. (d) Western blot analysis of α-SMA, E-cadherin, and β-actin in the kidneys from mice at 24 d post-surgery. (e) The quantitative results of panel c and d were shown, β-actin was used as the loading control (n = 6). **(f)** Representative images of E-cadherin immunofluorescence staining were shown in the kidney from mice at 24 d post-surgery. The lower panels are enlarged images of the boxed areas in the upper panels. White arrows indicate polarized distribution of E-cadherin in the basolateral membrane of tubules; red arrows indicate re-distribution of E-cadherin to the apical membrane of tubules. **(g,h)** Kidney lysates subjected to Co-IP with anti-SMAD2 or anti-SMAD3 antibody in the WT and *Sirt2 overexpression* mice at 24 d post-surgery, and western blotting using indicated antibodies (g). The quantitative results are shown in panel h (n = 6). **(i)** Western blot analyses of nuclear levels of SMAD2, SMAD3 and H3 in the fractions extracted from the kidney of the WT and *Sirt2 overexpression* at 24 d post-surgery. Quantitative results are shown in the right panel (n = 6). **(j)** qPCR analysis of the mRNA level of *Col1a1*, *Col1a2*, *Serpine1*, and *α-SMA* in the kidney of mice (n = 6). For all panels, data are presented as mean ± SD. **P* < 0.05, ***P* < 0.01, ****P* < 0.001 by one-way ANOVA with Bonferroni correction test.


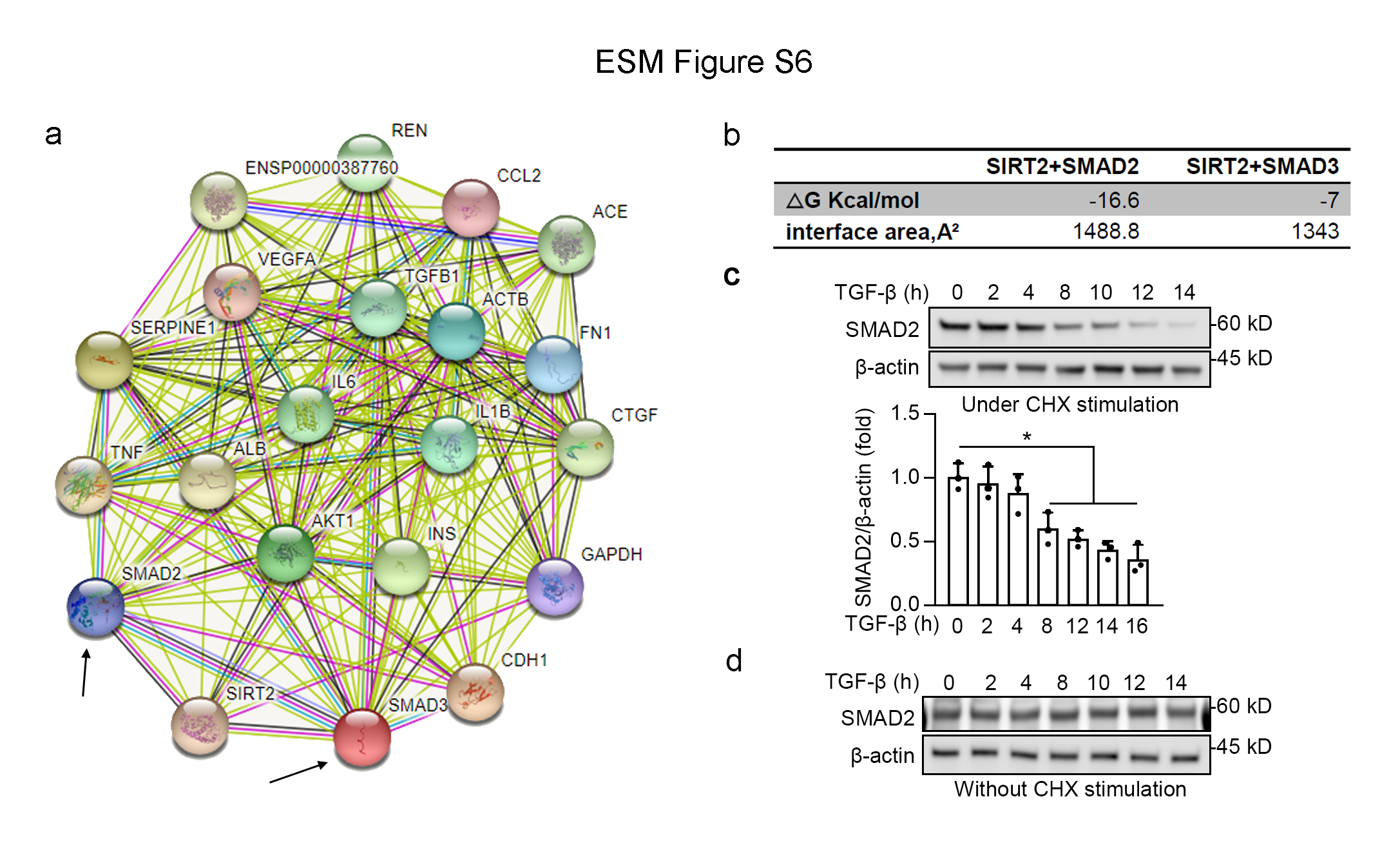


**Figure S6. The correlation between SMAD2, SMAD3 and SIRT2. (a)** Prediction of protein interactions using the STRING database showing two partner proteins, SIRT2 and SMAD2. **(b)** GRAMM-X was used to predict the binding capacity and interface area between proteins. **(c)** HK2 cells treated with TGF-β and/ CHX as indicated in figure, and the western blot analysis of SMAD2 and β-actin were shown in the upper panel, and the quantitative results are shown in the bottom panel (n = 3). **(d)** HK2 cells treated with TGF-β as indicated in figure, and the western blot analysis of SMAD2 and β-actin were shown. For all panels, data are presented as mean ± SD. **P* < 0.05 by one-way ANOVA with Bonferroni correction test.


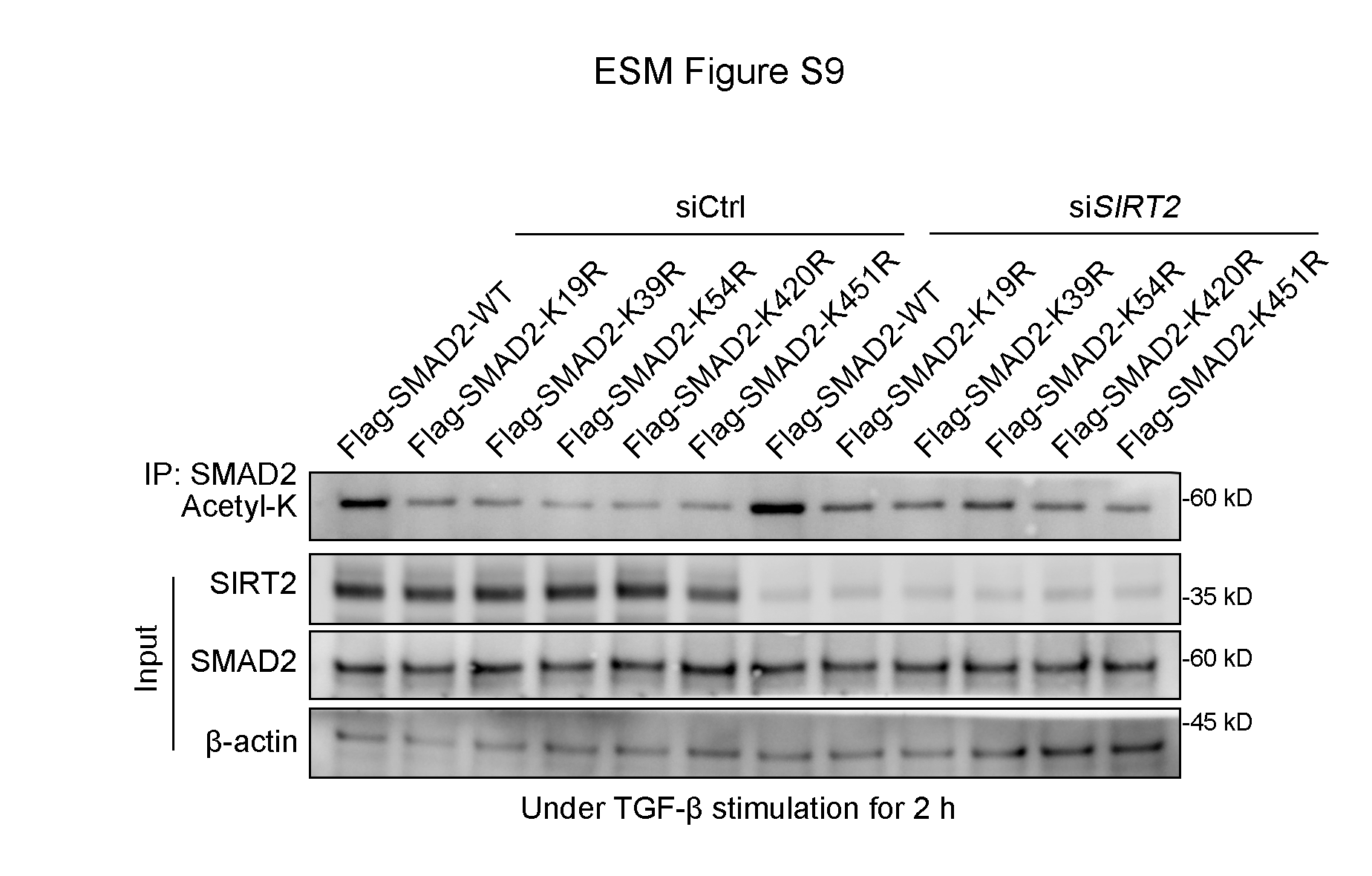


**Figure S7.** **SIRT2 deacetylated Lys451 on SMAD2.** The siCtrl or si*SIRT2* were transfected into HEK293T cells with either *SMAD2*-WT or different mutant plasmids including *SMAD2*-WT, *SMAD2*-K19R, *SMAD2*-K54R, *SMAD2*-K420R, and *SMAD2*-K451R for 24 h, followed by the treatment of TGF-β for 2 h. Lysates were immunoprecipitated with anti-Flag antibody and blotted with anti-acetyl-lysine antibody.


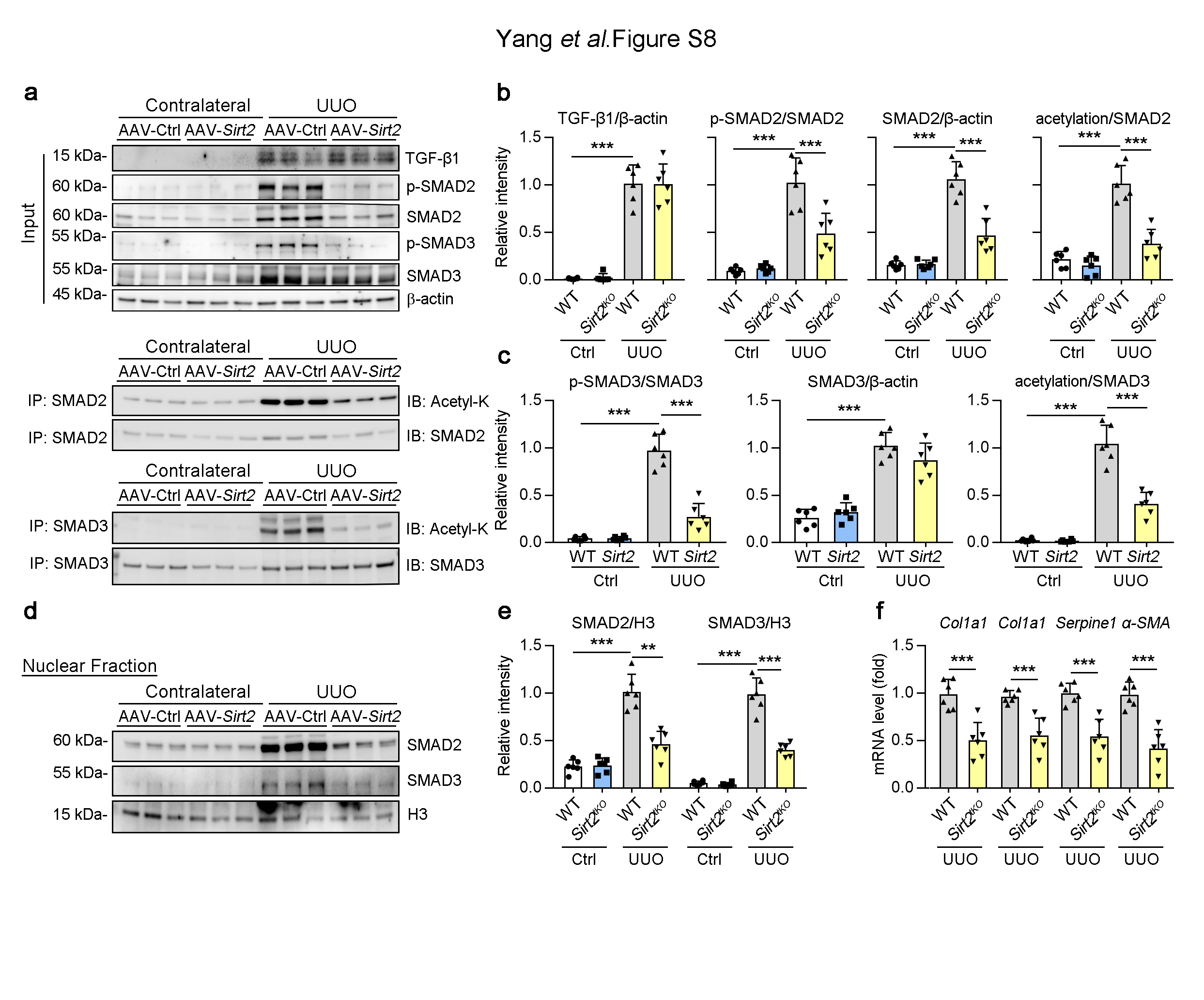


**Figure S8. Overexpression of SIRT2 in renal tubular epithelial cells inhibits the acetylation, phosphorylation, and nuclear accumulation of SMAD2 and SMAD3 in vivo.** AAV-Ctrl or AAV-*Ggt* (gamma-glutamyltransferase 1)-*Sirt2* transfected with C57BL/6J mice. After 2-week transfection, mice received UUO or sham surgery. **(a)** The kidney lysates were subjected to Co-IP with anti-SMAD2 or anti-SMAD3 antibody in the Sham and UUO mice at day 10 post-surgery, followed by western blotting using indicated antibodies (a), and the quantitative results were shown in the panel b and c (n = 6). **(d,e)** Western blot analyses (d) of nuclear levels of SMAD2 and H3 in the fractions extracted from the kidney of the WT and UUO mice at day 10 post-surgery and the quantitative results were shown in the panel e (n = 6). **(f)** qPCR analysis of the mRNA level of *Col1a1*, *Col1a2*, *Serpine1*, and *α-SMA* in the kidney of mice (n = 6). For all panels, data are presented as mean ± SD. **P* < 0.05, ***P* < 0.01, ****P* < 0.001 by one-way ANOVA with Bonferroni correction test.


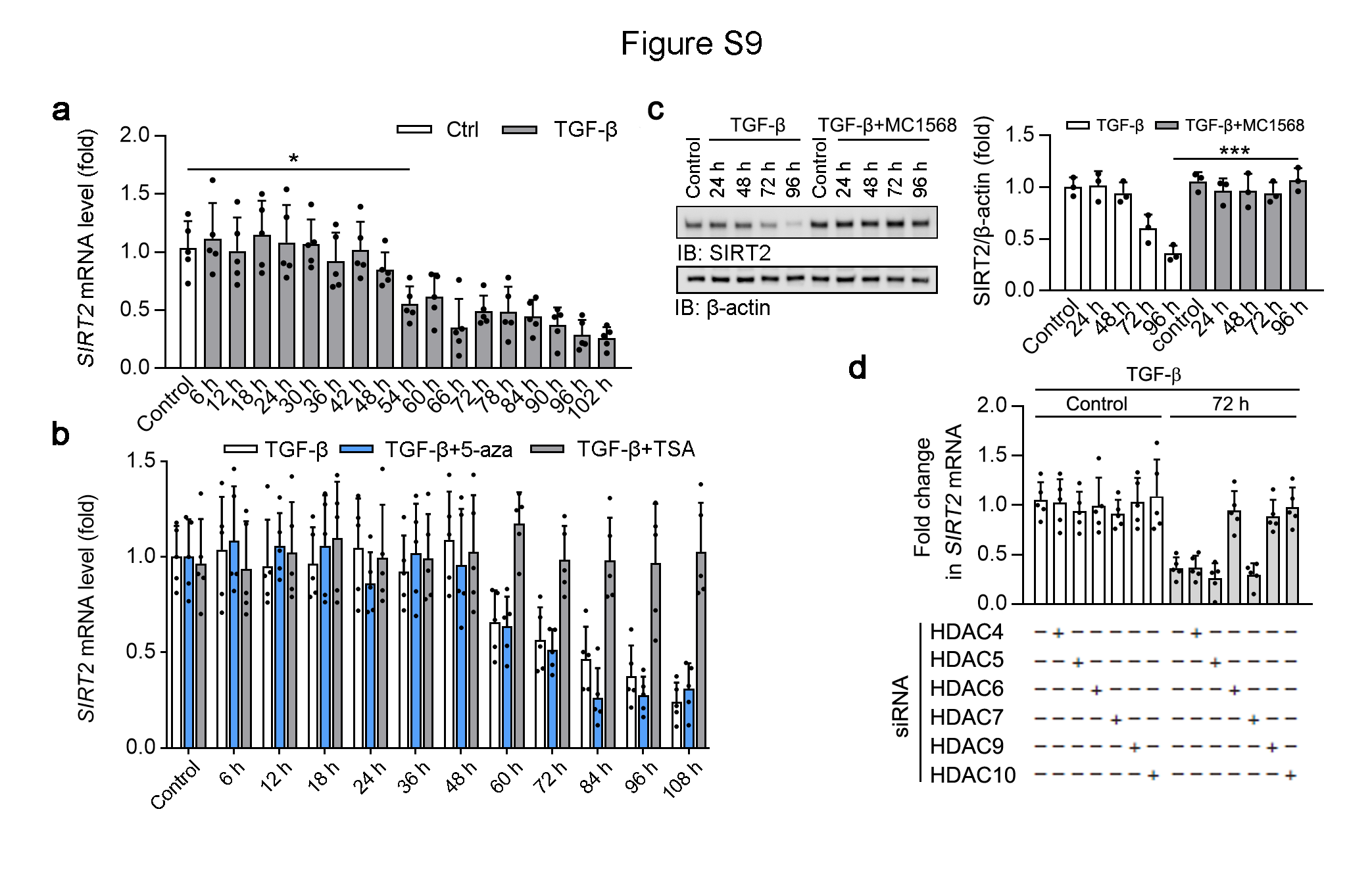


**Figure S9. Chronic stimulation of TGF-β inhibited SIRT2 expression through epigenetic modification. (a, b)** HK2 cells were incubated with TGF-β, 5-aza, or TSA as indicated, and the mRNA level of SIRT2 was determined by qRT-PCR, n=5. **(c)** Western blot analysis of the expression of SIRT2 and β-actin (left), and the quantification results are shown in the right panel (n = 3). **(d)** HK2 cells were transfected with siRNA-mediated knockdown of class II HDAC, followed by treatment with 2 ng/mL TGF-β, and the mRNA level of *SIRT2* in HK2 cells, and the mRNA level of *SIRT2* were detected by qPCR (n = 5). For all panels, data are presented as mean ± SD. **P* < 0.05, ***P* < 0.01, ****P* < 0.001 by one-way ANOVA with Bonferroni correction test.


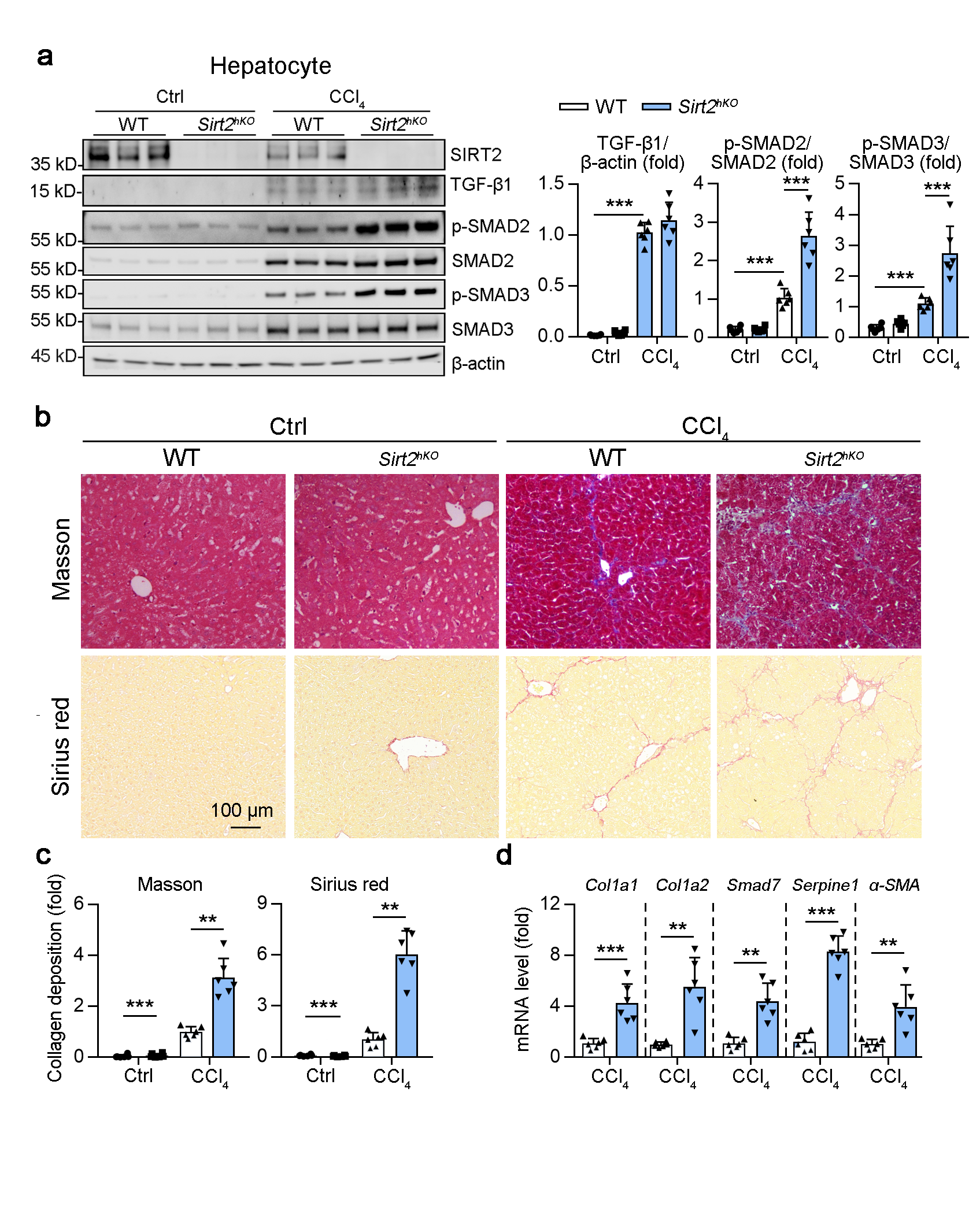


**Figure S10. Hepatocyte-specific SIRT2 deletion promoted hepatic fibrosis in CCL_4_ treated mice. (a to d)** Albumin-cre mice were crossed with *Sirt2^fl/fl^* to generate hepatocyte-specific *Sirt2* knockout mice (*Sirt2^hKO^*). (a) Western blot analysis of the phosphorylation of TGF-β1, SMAD2, SMAD3, and the expression of SMAD2, SMAD3 and SIRT2 and β-actin (left panel) in primary hepatocytes isolated from mouse liver, and the quantification results are shown in the right panel (n = 3). (b, c) Representative images of Sirius red and Masson’s trichrome staining were shown in panel b, and the collagen deposition (c) was quantified in the liver sections in 3 fields per mice at 100× magnification (n = 6). (d) The mRNA level of TGF-β regulated genes (*Col1a1, Col1a2, Smad7, Serpine1*, and *α-SMA*) were determined by qPCR in the liver of mice (n = 6). Key in (a) also applies to (c, d). For all panels, data are presented as mean ± SD. **P*< 0.05,***P* < 0.01, ****P* < 0.001, by unpaired Student’s t test (a, d). **P* < 0.05, ***P* < 0.01, ****P* < 0.001 by one-way ANOVA with Bonferroni correction test (c).

**Table S1. The interface residue pair between SIRT2 and SMAD2 predicted by HDOCK**

| Interface residues of SIRT2 | Interface residues of SMAD2 | Corresponding distances |
| --- | --- | --- |
| 114A | 283A | 2.555 |
| 114A | 284A | 3.529 |
| 116A | 284A | 4.273 |
| 117A | 283A | 3.873 |
| 120A | 283A | 2.736 |
| 120A | 284A | 4.141 |
| 121A | 432A | 3.758 |
| 122A | 283A | 4.391 |
| 122A | 430A | 4.062 |
| 122A | 431A | 4.655 |
| 122A | 432A | 1.071 |
| 122A | 433A | 4.262 |
| 123A | 283A | 4.913 |
| 123A | 432A | 4.743 |
| 125A | 430A | 4.291 |
| 126A | 430A | 4.075 |
| 126A | 433A | 4.146 |
| 163A | 265A | 3.948 |
| 180A | 458A | 4.958 |
| 192A | 459A | 4.842 |
| 198A | 407A | 4.721 |
| 201A | 403A | 2.488 |
| 201A | 404A | 2.909 |
| 201A | 407A | 1.718 |
| 202A | 403A | 2.182 |
| 202A | 460A | 3.351 |
| 235A | 280A | 4.705 |
| 235A | 283A | 4.596 |
| 236A | 280A | 3.205 |
| 236A | 283A | 4.886 |
| 236A | 415A | 3.118 |
| 236A | 437A | 3.668 |
| 236A | 439A | 3.166 |
| 237A | 280A | 4.294 |
| 237A | 415A | 3.549 |
| 237A | 439A | 3.594 |
| 238A | 413A | 2.864 |
| 238A | 415A | 3.821 |
| 238A | 439A | 2.472 |
| 238A | 441A | 2.351 |
| 239A | 409A | 3.189 |
| 239A | 441A | 3.751 |
| 240A | 406A | 4.377 |
| 240A | 409A | 3.743 |
| 241A | 409A | 3.009 |
| 241A | 412A | 4.882 |
| 241A | 446A | 3.522 |
| 241A | 449A | 2.995 |
| 241A | 450A | 4.959 |
| 242A | 450A | 2.889 |
| 242A | 453A | 4.41 |
| 242A | 454A | 3.976 |
| 244A | 276A | 3.775 |
| 244A | 441A | 2.601 |
| 244A | 443A | 3.071 |
| 244A | 446A | 2.347 |
| 244A | 447A | 4.113 |
| 245A | 268A | 3.31 |
| 245A | 446A | 3.435 |
| 245A | 447A | 2.732 |
| 245A | 450A | 2.349 |
| 245A | 451A | 4.598 |
| 246A | 447A | 4.541 |
| 246A | 450A | 3.802 |
| 248A | 269A | 2.692 |
| 248A | 270A | 4.45 |
| 248A | 271A | 1.268 |
| 248A | 272A | 3.872 |
| 248A | 273A | 3.684 |
| 248A | 443A | 3.603 |
| 248A | 447A | 3.467 |
| 249A | 267A | 3.662 |
| 249A | 268A | 3.659 |
| 249A | 269A | 3.276 |
| 249A | 447A | 4.808 |
| 252A | 269A | 2.166 |
| 252A | 270A | 4.596 |
| 253A | 265A | 4.491 |
| 253A | 267A | 3.401 |
| 267A | 285A | 3.438 |
| 267A | 287A | 4.911 |
| 267A | 288A | 4.473 |
| 267A | 289A | 3.233 |
| 268A | 285A | 4.508 |
| 268A | 289A | 3.664 |
| 268A | 291A | 4.69 |
| 271A | 291A | 3.642 |
| 299A | 303A | 4.231 |
| 299A | 305A | 2.621 |
| 299A | 315A | 3.133 |
| 300A | 288A | 4.317 |
| 300A | 305A | 1.708 |
| 300A | 306A | 3.365 |
| 300A | 307A | 4.865 |
| 301A | 288A | 2.989 |
| 301A | 289A | 4.769 |
| 302A | 288A | 3.409 |

The interface residues within 5.0 Å from their interacting partner or each other, and the corresponding distances. A: amino acid.

Table S2. The primers sequences used in qPCR analysis

| Gene | Forward | Backward |
| --- | --- | --- |
| *homo-SIRT2*  (ID:[22933](http://www.ncbi.nih.gov/entrez/query.fcgi?db=gene&cmd=Retrieve&dopt=summary&list_uids=22933)) | CACGCAGAACATAGATACCCTG | CAGTGTGATGTGTAGAAGGTGC |
| *mus-α-SMA*  (ID: 11475) | CCCAACTGGGACCACATGG | GGTACATGCGGGGGACATTGAAG |
| *mus-E-cadherin*  (ID: 12550) | CAGGTCTCCTCATGGCTTTGC | CTTCCGAAAAGAAGGC  TGTC |
| *homo-FN1*  (ID: 2335) | GAGAATAAGCTGTACCATCGCAA | CGACCACATAGGAAGTCCCAG |
| *homo-CTGF*  (ID: 1490) | AAAAGTGCATCCGTACT  CCCA | CCGTCGGTACATACTCC  ACAG |
| *homo-α-SMA*  (ID: 58) | GGCATTCACGAGACCACCTAC | CGACATGACGTTGTTGGCATAC |
| *mus-Col1a1*  (ID: 12842) | GCTCCTCTTAGGGGCCACT | CCACGTCTCACCATTGGGG |
| *mus- Col1a2*  (ID: 12843) | GTAACTTCGTGCCTAGCAACA | CCTTTGTCAGAATACTGAGCAGC |
| *mus-Smad7*  (ID: 17131) | GGCCGGATCTCAGGCATTC | TTGGGTATCTGGAGTAAGGAGG |
| *mus-Serpine1*  (ID: 18787) | TTCAGCCCTTGCTTGCCTC | ACACTTTTACTCCGAAGTCGGT |
| *mus-TGF-β*  (ID: 21803) | CTCCCGTGGCTTCTAGTGC | GCCTTAGTTTGGACAGGATCTG |
| *mus-β-actin*  (ID: 11461) | GGCTGTATTCCCCTCCATCG | CCAGTTGGTAACAATGCCATGT |
| *h-β-actin*  (ID: 60) | CATGTACGTTGCTATCCAGGC | CTCCTTAATGTCACGCACGAT |
