# Supplementary figures and images for: SIRT2 alleviated renal fibrosis by deacetylating SMAD2 and SMAD3 in renal tubular epithelial cells
Source: Cell Death Dis. 2023 Sep 30;14(9):646. doi: 10.1038/s41419-023-06169-1 (PMC10542381; doi:10.1038/s41419-023-06169-1)

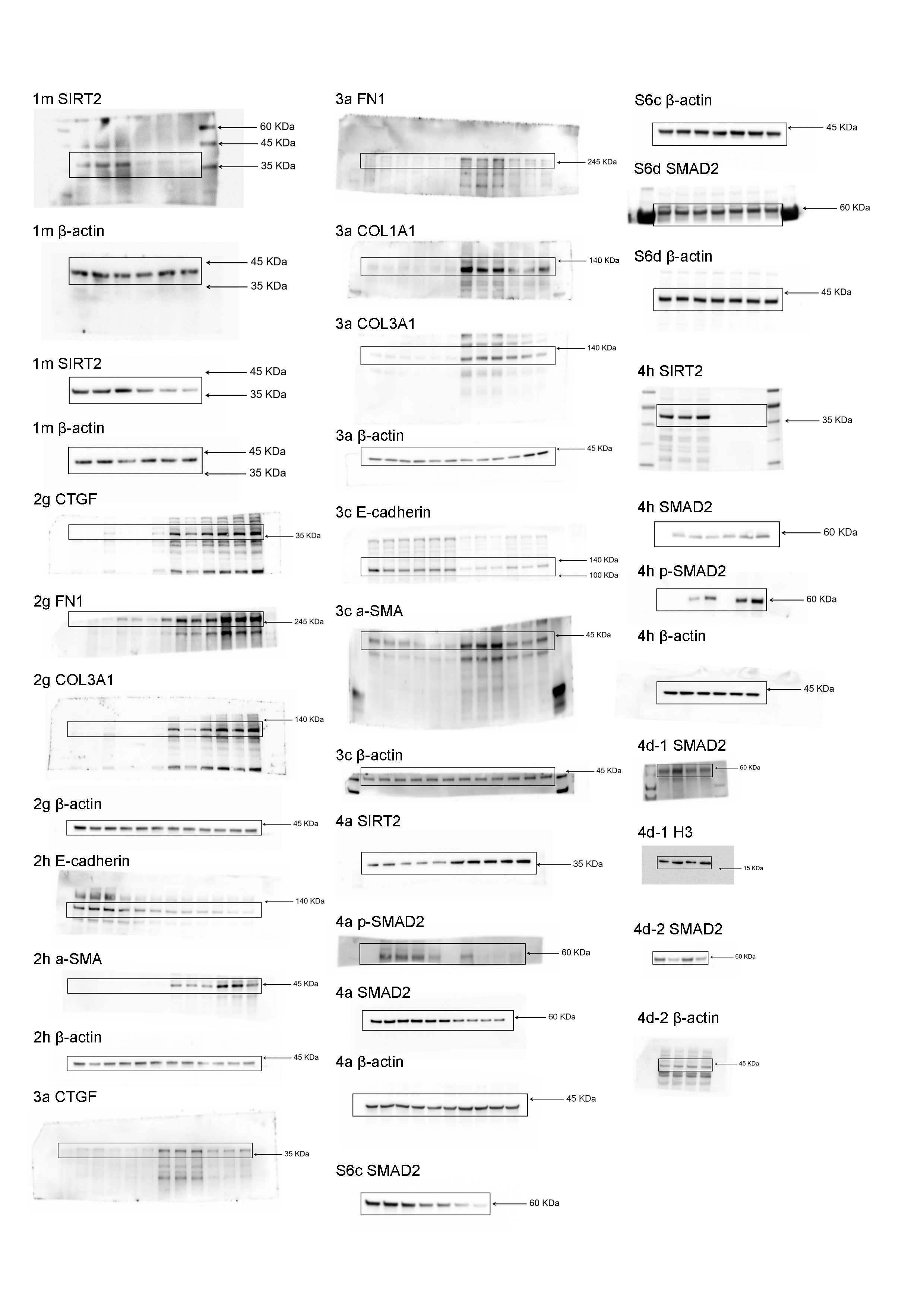

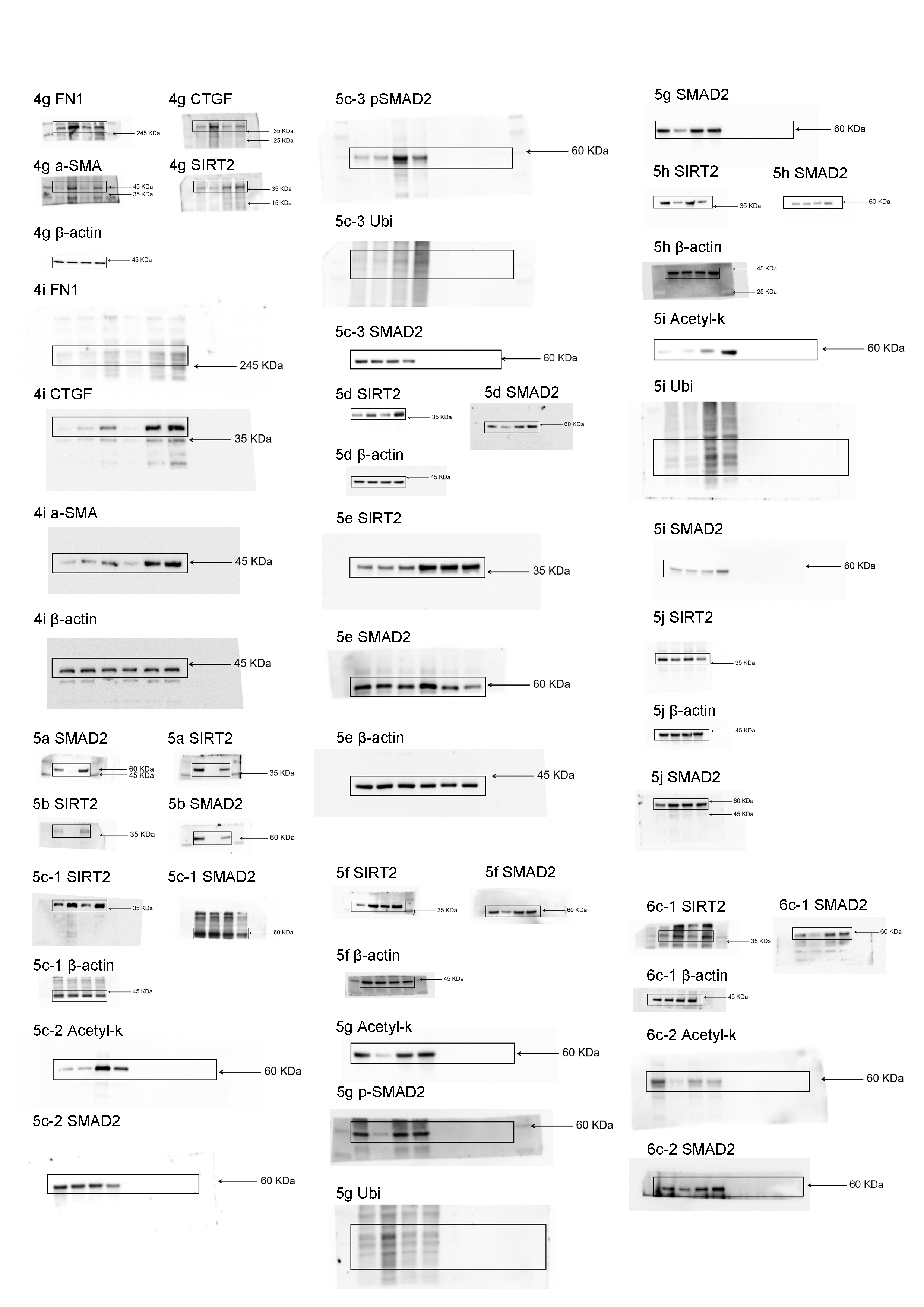

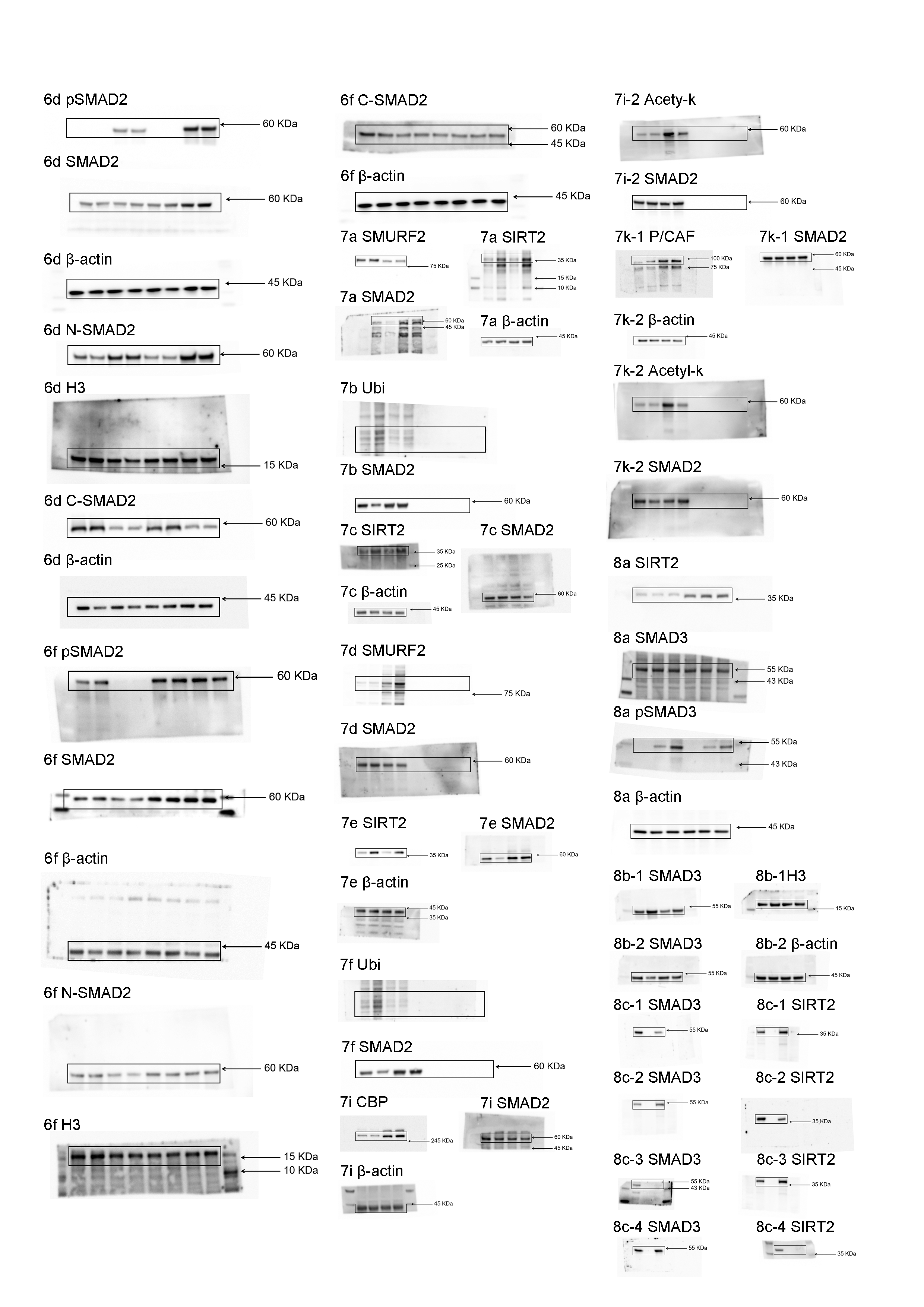

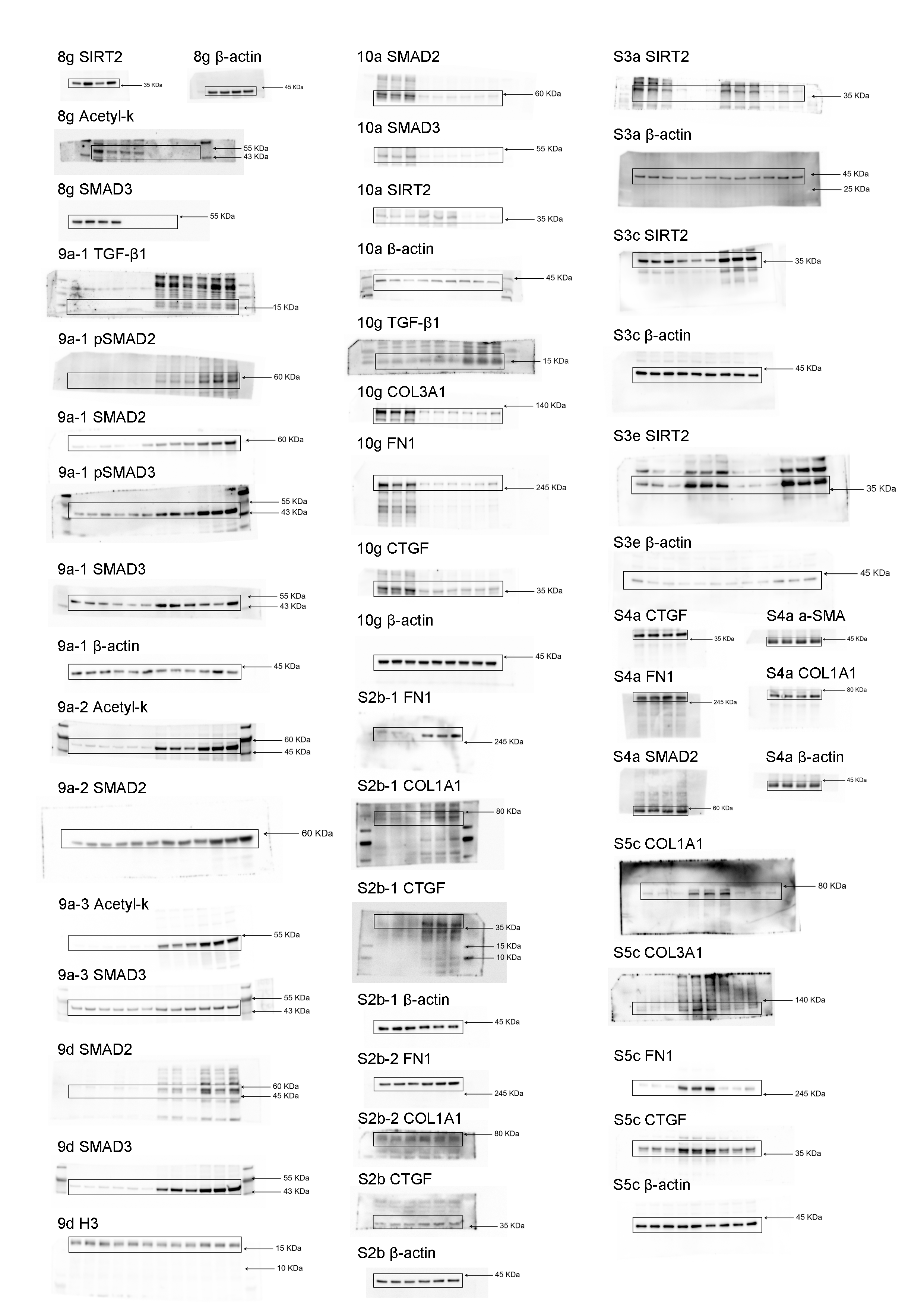

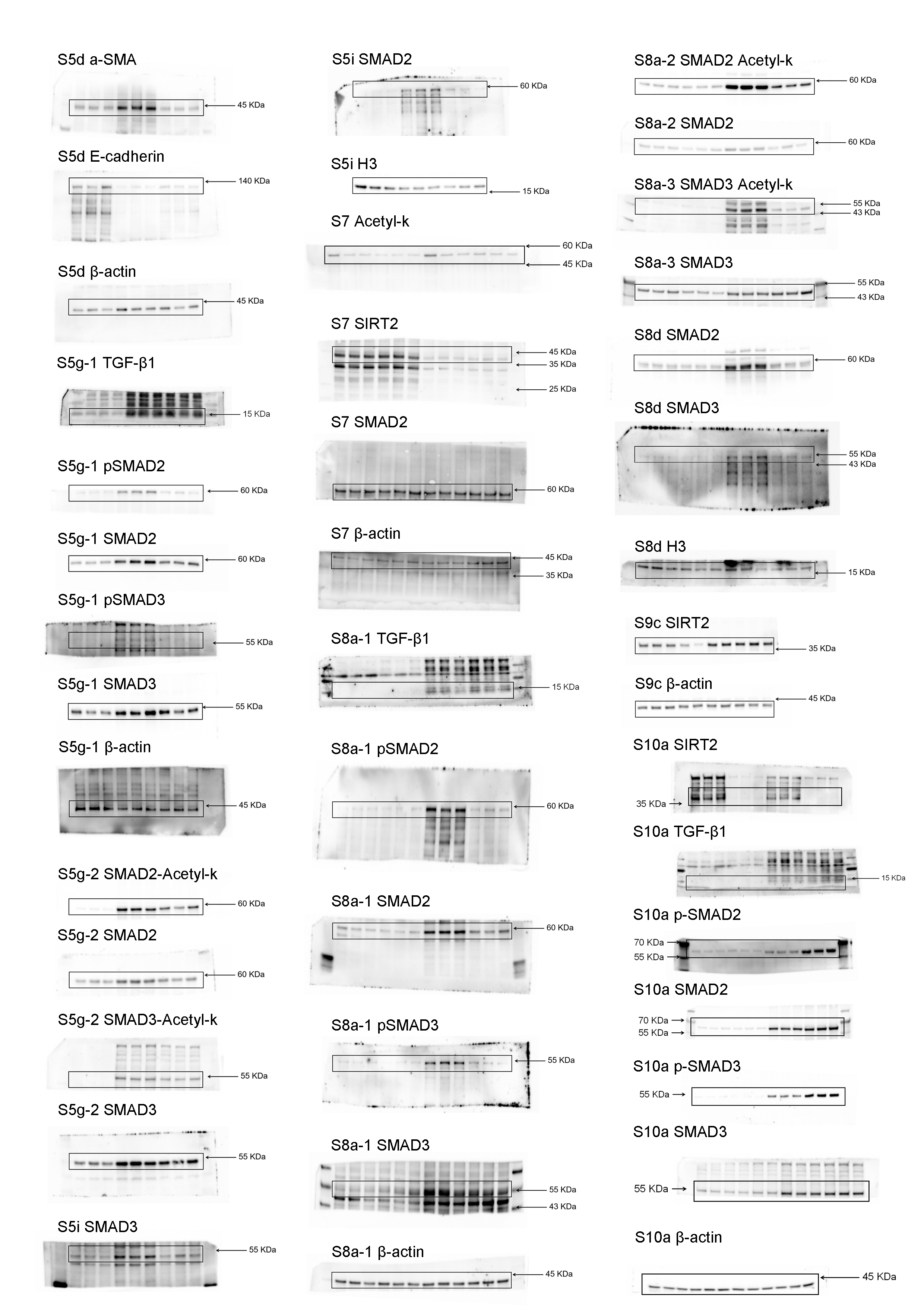

Supplement: Supplementary file 2 — Full and uncropped western blots [file 41419_2023_6169_MOESM2_ESM.docx]
